# Statins Regulate Stem Cell Growth Factor‐β to Balance Osteogenesis and Adipogenesis in Mesenchymal Stem Cells, Endowing Anti‐Osteonecrosis Effects

**DOI:** 10.1111/jcmm.70967

**Published:** 2025-11-26

**Authors:** Fangzhou Fan, Yu Chen, Weiyan Peng, Wenlong Yan, Hao Tan, Chengxuan Zhang, Siyu Tan, Qian Xiao, Yuan Gao, Jian Zhang, Lei Liu, Chengjie Lian

**Affiliations:** ^1^ Department of Orthopedics, Chongqing Municipal Health Commission Key Laboratory of Musculoskeletal Regeneration and Translational Medicine the First Affiliated Hospital of Chongqing Medical University Chongqing China; ^2^ Chongqing Key Laboratory of Molecular Oncology and Epigenetics, Department of Breast and Thyroid Surgery The First Affiliated Hospital of Chongqing Medical University Chongqing China; ^3^ Department of Geriatrics The First Affiliated Hospital of Chongqing Medical University Chongqing China; ^4^ Department of Orthopaedics, Sports Injury Division Fujian Medical University Union Hospital Fuzhou China

**Keywords:** mendelian randomisation, mesenchymal stem cells, osteonecrosis, statins, stem cell growth factor‐β

## Abstract

Dyslipidaemia has been implicated in osteonecrosis through some clinical studies. However, a direct causal relationship between hyperlipidaemia and osteonecrosis remains unconfirmed, and whether lipid‐lowering agents could be used to treat osteonecrosis remains unclear. This study aimed to investigate the causal role of lipid traits in osteonecrosis using Mendelian randomisation (MR) analysis, assess the potential effects and mechanisms of lipid‐lowering drug targets on osteonecrosis risk and validate these findings through experimental approaches. Genome‐wide association study (GWAS) data were used to analyse lipid traits, drug targets and FinnGen osteonecrosis. Statin effects were further studied in a rat model of steroid‐induced osteonecrosis and in vitro cell models. MR analysis revealed a significant association between LDL‐C and increased osteonecrosis risk. Genetic mimicry of HMGCR inhibitors was associated with reduced osteonecrosis risk, which was validated through colocalisation. Stem cell growth factor‐β (SCGF‐β) was identified as a mediator of 21.3% of HMGCR inhibitors' effect on osteonecrosis risk. Further studies confirmed simvastatin's alleviating effect on SONFH, suggesting that simvastatin promotes osteogenesis and inhibits adipogenesis of mesenchymal stem cells (MSCs), partly mediated by SCGF‐β upregulation, which activates the Wnt signalling pathway. Our findings supported dyslipidaemia as a causal factor for osteonecrosis, highlighting HMGCR as a promising therapeutic target.

**Abbreviations**
CAD: coronary artery disease
CTACK: cutaneous T cell‐attracting chemokine
eQTL: expression quantitative trait loci
GRS: genetic risk scores
GSMR: generalised summary‐data‐based Mendelian randomisation
GWAS: genome wide association study
IVs: instrumental variables
IVW: inverse‐variance weighted
LD: linkage disequilibrium
LDL‐C: low‐density lipoprotein cholesterol
MR: Mendelian randomisation
MSCs: mesenchymal stem cells
ONFH: osteonecrosis of the femoral head
PCSK9: proprotein convertase subtilisin/kexin type 9
RAPS: robust adjustment profile score
SCGF‐β: stem cell growth factor‐beta
SNPs: single nucleotide polymorphisms
TC: total cholesterol
TG: triglycerides
TRAIL: tumour necrosis factor‐related apoptosis‐inducing ligand

## 1 Introduction

Osteonecrosis is a serious skeletal disease that can occur in any bone, but the most affected site is the femoral head. Osteonecrosis of the femoral head (ONFH) includes both traumatic and non‐traumatic forms. Common risk factors for non‐traumatic osteonecrosis of the femoral head (NONFH) include excessive alcohol consumption, use of steroids, chemotherapy, immunosuppressive drugs and sickle cell anaemia [1]. According to 2010 population data from China, about 8 million people aged 15 and older in the general Chinese population were affected by NONFH [2]. In the United States, there are approximately 10,000–20,000 new cases of NONFH each year [3]. About 86% of new NONFH patients are in the early to middle stages of the disease progression (ARCO stages I, II, III) [2]. ONFH is a progressive disease, and once it advances to the collapse stage (ARCO stage IV), hip replacement surgery is inevitably required for the patients [4]. However, at present, there is no clearly established optimal treatment for pre‐collapse ONFH [5].

Local ischaemia caused by impaired blood flow is the primary pathogenesis of ONFH. Hyperlipidaemia, a condition characterised by abnormally elevated levels of lipids (such as cholesterol and triglycerides) in the blood, is considered an important contributing factor for impaired blood supply in ONFH. Hyperlipidaemia can lead to ischemia in the microcirculation through various mechanisms, including increasing blood viscosity, promoting thrombosis formation, impairing endothelial function, triggering inflammation and oxidative stress, inducing vasoconstriction and increasing platelet activity [6]. Yang's study analysed the serum lipid levels of 182 ONFH patients and 179 healthy controls, showing significantly higher levels of triglycerides (TG), total cholesterol (TC) and low‐density lipoprotein cholesterol (LDL‐C) in the ONFH group compared to the control group [7]. Steroids and alcohol are two major risk factors for NONFH, but they can also cause varying degrees of hyperlipidaemia [8]. Xu's study found that non‐steroid and non‐alcohol‐related ONFH patients also had elevated levels of triglycerides and total cholesterol [9]. Similarly, the levels of TC, TG, LDL and LDL/HDL were significantly higher in patients with idiopathic ONFH compared to the control group [10]. There appears to be a certain correlation between hyperlipidaemia and osteonecrosis; however, till now, no clinical or animal study has been conducted to provide evidence of a direct causal relationship between hyperlipidaemia and osteonecrosis.

Considering the potential disease‐causing role of hyperlipidaemia in ONFH, the lipid‐lowering drugs (e.g., statins and fibrates) have been tested to treat ONFH. Several animal studies have shown that the use of statins, the lipid‐lowering drugs, effectively reduces the incidence of steroid‐induced osteonecrosis and lowers blood lipid levels as well [11, 12]. However, the pharmacological effects of statins are complex, and the mechanisms underlying their anti‐osteonecrosis effects remain largely unknown. Jiang's study indicated that simvastatin may prevent SONFH by inhibiting PPARγ expression and activating the Wnt signalling pathway [13], while Sakamoto's study suggested that simvastatin may prevent SONFH by inhibiting the expression and secretion of plasminogen activator inhibitor‐1 (PAI‐1) in bone marrow adipocytes [14]. In addition, retrospective clinical studies have shown a reduced incidence of ONFH in patients using statins alongside steroids [15, 16]. Furthermore, fenofibrate has been shown to be able to control hypertriglyceridemia successfully induced by dexamethasone and be associated with a reduced frequency and severity of osteonecrosis [17]. Therefore, it is necessary to determine whether lipid‐lowering drugs are beneficial for the treatment of osteonecrosis and reveal the underlying molecular mechanisms.

Mendelian randomisation (MR) utilises genetic variations closely associated with exposure as instrumental variables (IVs) to infer potential causal relationships between two phenotypes [18]. This technique can minimise confounding and avoid reverse causation bias to a great extent due to the fact that the association between genes and diseases is not influenced by common confounding factors such as environmental, socioeconomic factors and individual behaviours [19, 20]. With advancements in research technology, targeted drug MR analysis has become a powerful tool for investigating the strong link between protein‐coding genes and disease risk. By using these single‐nucleotide polymorphisms (SNPs) as IVs, targeted drug MR analysis provides valuable insights into potential causal relationships between specific genes and disease risk [21, 22]. Currently, the MR analysis method has been widely applied. A classic example is that individuals harbouring genetic variations linked to reduced levels of LDL‐C in the PCSK9 gene exhibited a decreased occurrence of coronary heart disease. This finding laid the foundation for the development of proprotein convertase subtilisin/kexin type 9 (PCSK9) inhibitors, and subsequent RCTs confirmed the effectiveness of PCSK9 inhibitors [23]. Now, MR analysis has also been used to predict the impact of drug targets on musculoskeletal diseases such as osteoporosis and arthritis [24, 25].

**FIGURE 1:**
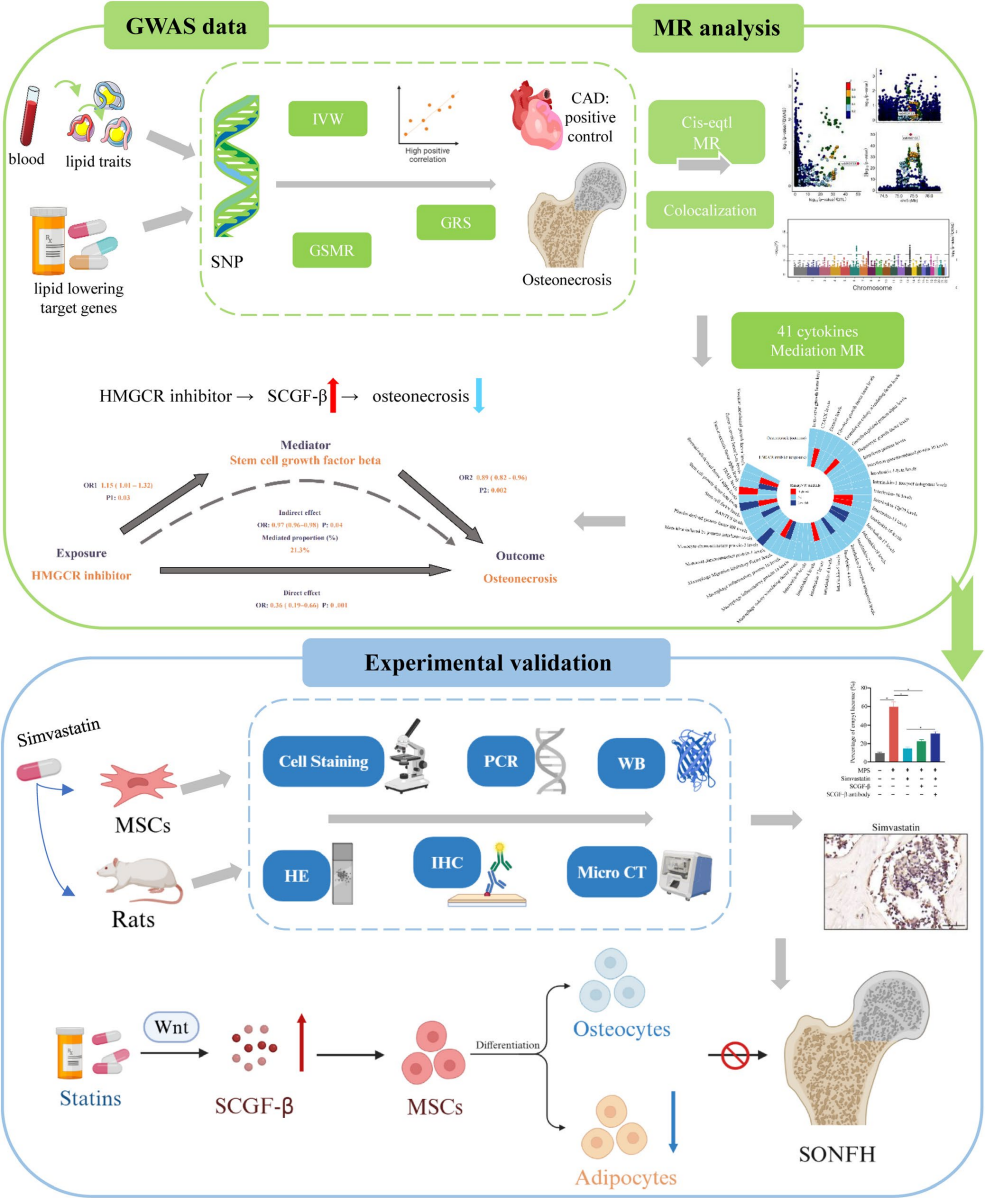
The flowchart of study design. ANGPTL3, Angiopoietin‐Like 3; GRS, genetic risk scores; GSMR, generalised summary‐data‐based mendelian randomisation; HE, Haematoxylin–Eosin staining; HMGCR, HMG‐CoA reductase; IHC, immunohistochemistry; LDL‐C, low‐density lipoprotein cholesterol; MR, mendelian randomisation; MSCs, mesenchymal stem cells; PCR, polymerase chain reaction; SCGF‐β, stem cell growth factor‐β; SNP, single nucleotide polymorphisms; SONFH, steroid‐induced osteonecrosis of the femoral head; WB, western Blot.

In this study, we adopted MR analyses to determine the effects of lipid traits on osteonecrosis and explore the potential effects and mechanisms of lipid‐lowering drug targets on osteonecrosis. Additionally, we validated the anti‐osteonecrosis effect of simvastatin and studied the underlying molecular mechanisms, using the rat SONFH model and in vitro cell model as well. The study design is outlined in Figure 1. Our research could potentially provide new insights into osteonecrosis aetiology, offer novel therapeutic targets and may improve patient prognosis.

## 2 Materials and Methods

### 2.1 Genetic Variant Selection

Independent genetic variants linked to six lipid traits (TC, TG, HDL‐C, LDL‐C, ApoA1 and ApoB) were identified in a comprehensive meta‐analysis conducted by the Global Lipids Genetics Consortium (GLGC) [26] and UK Biobank [27]. These variants were found to be statistically significant at a genome‐wide level (*p* < 5 × 10^−8^), with independent associations observed using specific thresholds for linkage disequilibrium (LD) (*r* ^2^ < 0.001) and physical distance (10,000 kb). The classification of these medications and their corresponding target genes was based on the latest expert consensus and guidelines for therapies aimed at reducing lipids [28, 29], as summarised in Table 1. Genes encoding pharmacological targets of these medications were identified using the DrugBank database (https://go.drugbank.com/) and relevant reviews [30, 31, 32]. A total of 11 target genes were identified, and genetic variants within these genes that encode protein targets of drugs used to lower lipid levels (cis‐variants) were extracted from the genome‐wide association study (GWAS) summary data provided by the GLGC [26]. We utilised publicly accessible eQTL data from the Genotype‐Tissue Expression project (GTEx‐V8) [33] and the eQTLGen Consortium (https://eqtlgen.org/) [34] to investigate drug targets that exhibited significance in relation to osteonecrosis risk, according to the MR analysis. This MR research adhered to the reporting guidelines of Strengthening the Reporting of Observational Studies in Epidemiology‐Mendelian Randomisation (Table S1). A comprehensive overview of these datasets can be found in Table S2.

**TABLE 1:** Lipid‐lowering drug classes, substances and target genes.

Drug effect	Drug class	Drug target	Encoding genes	Gene region	Drug substance	Eligible IVs
LDL‐C	Key Modulator	LDL Receptor	LDLR	CHR:19:11,200,038‐11,244,492	—	Yes
	HMGCR inhibitors	HMG‐CoA reductase	HMGCR	CHR:5:74,632,154‐74,657,929	Atorvastatin Rosuvastatin etc.	Yes
	ACLY inhibitors	ATP‐citrate synthase	ACLY	CHR:17:40,023,161‐40,086,795	Bempedoic acid	No
	PCSK9 inhibitors	Proprotein Convertase Subtilisin/Kexin Type 9	PCSK9	CHR:1:55,505,221‐55,530,525	Evolocumab Alirocumab	Yes
	TC absorption inhibitors	Niemann‐Pick C1‐like 1	NPC1L1	CHR:7:44,552,134‐44,580,914	Ezetimibe	Yes
	ASO targeting ApoB mRNA	Apo B100	APOB	CHR:2:21,224,301‐21,266,945	Mipomersen	Yes
	ASO targeting CETP mRNA	Cholesteryl Ester Transfer Protein	CETP	CHR:16:56,995,762‐57,017,757	Torcetrapib	Yes
	Bile acid sequestrants	Bile acids	—	—	Cholestyramine Colestipol	No
TG	Key Modulator	Lipoprotein Lipase	LPL	CHR:8:19,759,228‐19,824,769	—	Yes
	Fibrates	Peroxisome Proliferator‐Activated Receptor‐alpha	PPARA	CHR:22:46,546,424‐46,639,653	Fenofibrate Gemfibrozil	No
	ANGPTL3 inhibitors	Angiopoietin‐related protein 3	ANGPTL3	CHR:1:63,063,158‐63,071,830	Evinacumab	Yes
	ASO targeting ApoC‐III mRNA	Apo C‐III	APOC3	CHR:11:116,700,422‐116,703,788	Volanesorsen	Yes

### 2.2 Data Sources

The outcome was osteonecrosis. The GWAS summary of osteonecrosis data from the FinnGen R9 consortium includes 1385 cases and 358,014 controls from European populations, with osteonecrosis diagnosed using ICD‐10 M87 and ICD‐97334 codes [35]. And our MR analysis also utilised obtaining summary statistics of circulating cytokines from publicly available GWAS, which involved a total of 8293 participants and aimed to investigate the genetic associations between 41 circulating cytokines [36].

### 2.3 Mendelian Randomisation

The overall estimate of the causal effect of genetically proxied circulating lipid traits on osteonecrosis and genetically proxied lipid‐lowering treatment on osteonecrosis was obtained using the inverse‐variance weighted (IVW) method, which incorporated fixed/random effects (Figure S1). We assessed instrument relevance via *F*‐statistics, considering SNPs with *F* ≥ 10 resistant to weak‐instrument bias. In order to strengthen the credibility of the causal effects attributed to gene variants, we conducted positive control analyses by considering the well‐established benefits of lipid‐lowering drugs in coronary artery disease (CAD). We obtained summary statistics for CAD from Coronary Artery Disease [37].

For drug targets that exhibited significant association with osteonecrosis risk, we performed colocalisation analysis to test the exclusion restriction assumption [38]. This analysis estimated the probability (PP.H4) that SNPs jointly associated with the drug target and osteonecrosis are influenced by a shared causal variant at the locus. Drug targets demonstrating strong colocalisation with osteonecrosis (PP.H4 > 0.80) were regarded as potential target genes [38, 39].

In order to corroborate the aforementioned MR findings, a secondary analysis was conducted utilising the Genetic risk scores (GRS) and Generalised Summary‐data‐based MR (GSMR) approach [40]. The heterogeneity and pleiotropy among SNPs were assessed using Cochran's *Q* test and the MR‐Egger intercept test. We conducted leave‐one‐out analyses to evaluate the impact of removing a single influential SNP on overall estimates of causal effects in drug‐target proxies. Given that the genetic instruments selected as proxies for the drug target exhibited weak LD (*r* ^2^ < 0.3), we calculated LD correlation between genetic variants using LDlinkR (https://github.com/CBIIT/LDlinkR) and adjusted for LD structure in sensitivity analyses. To ensure the robustness of our findings, more stringent LD thresholds (*r* ^2^ < 0.1, *r* ^2^ < 0.01 and *r* ^2^ < 0.001) were applied to test significant drug‐target MR associations observed initially [41, 42].

A two‐step MR approach was employed to explore both the direct and indirect effects of exposure (lipid‐lowering drug targets) on the outcome (osteonecrosis), thereby enabling the identification of potential mediators (circulating cytokines) within causal associations. In the first step, we assessed the causal association between exposure and the mediator, and estimated the causal effect (β1). In the second step, we evaluated the causal association between the mediator and outcome, which was quantified as β2. The overall causal effect of exposure on outcome was defined as β; we further computed the indirect effect as (β1 × β2), the direct effect as (β − β1 × β2) and the mediation proportion as (β1 × β2)/β.

### 2.4 Rat Model

Healthy male Sprague–Dawley (SD) rats aged 12 weeks old with a body weight of 350–450 g were used for the animal experiments in this study. All the animal experiments were approved by the Chongqing Medical University Animal Care and Use Committee. The SD rats were purchased from the Animal Centre of Chongqing Medical University. All the animal experiments were performed in an SPF‐grade animal laboratory. The animals were housed under temperature (22°C ± 2°C) and humidity (around 60%) conditions with a standard light (12 h light/dark) cycle.

The first part of the animal experiments consists of two groups: (1) NC group (rats were treated with oral administration of saline); (2) Simvastatin group (rats were treated with oral administration of Simvastatin). 20 rats were randomly divided into these two groups. Simvastatin (Sigma‐Aldrich, St. Louis, MO, USA) (25 mg/kg/d) dissolved in saline or an equal volume of saline was administered for 3 weeks. After the treatment, the rat serum was collected and stored at −80°C, and then the rats were euthanised by carbon dioxide asphyxiation, and the femoral heads were collected and fixed with 4% paraformaldehyde.

The second part contains five groups: (1) NC group (rats as negative control were treated with oral administration of saline, intramuscular injection of saline and femoral intramedullary injection of saline); (2) MPS group (rats were treated with oral administration of saline, intramuscular injection of MPS and femoral intramedullary injection of saline); (3) MPS + Simvastatin group (rats were treated with oral administration of Simvastatin, intramuscular injection of MPS and femoral intramedullary injection of saline); (4) MPS + SCGF‐β group (rats were treated with oral administration of saline, intramuscular injection of MPS and femoral intramedullary injection of purified SCGF‐β protein); (5) MPS + Simvastatin + SCGF‐β antibody group (rats were treated with oral administration of Simvastatin, intramuscular injection of MPS and femoral intramedullary injection of SCGF‐β neutralising antibody). 10 rats were randomly assigned to each group. The simvastatin regimen was the same as in the first part, beginning with MPS injection. MPS (Pfizer Inc., New York, USA) (20 mg/kg/d) or an equal volume of saline was intramuscularly injected into rats once a day, on the first 3 days of every week, for 3 weeks. Purified SCGF‐β protein, SCGF‐β neutralising antibody or an equal volume of saline was injected into the femoral medullary cavity after using MPS for a week. The intramedullary injection protocol refers to the team's previous article [43]. After treatment for 8 weeks, the rats were euthanised by carbon dioxide asphyxiation, and the femoral heads were collected and fixed in 4% paraformaldehyde.

### 2.5 Cell Culture and Treatment

Human MSCs were obtained from Shanghai Zhong Qiao Xin Zhou Biotechnology and cultured in regular culture medium (DMEM with 10% FBS and 1% penicillin/streptomycin). MSCs were cultured with 200 μM simvastatin (HY‐17502, MCE, New Jersey, USA), 10 μg/mL SCGF‐β recombinant protein (ab9836, Abcam, Cambridge, UK), 100 μg/mL SCGF‐β neutralising antibody (ab9835, Abcam) or 5 μM ICG‐001(HY‐14428, MCE) for 48 h. For osteogenic induction, MSCs were cultured with osteogenic medium supplemented with 50 μM ascorbic acid (Sigma‐Aldrich), 10 nM dexamethasone (Sigma‐Aldrich), and 10 mM β‐glycerophosphate (Sigma‐Aldrich). For adipogenic induction, MSCs were treated with adipogenic medium containing 10 μg/mL insulin (Sigma‐Aldrich), 1 μM dexamethasone (Sigma‐Aldrich) and 0.5 μM isobutylmethylxanthin (Sigma‐Aldrich).

Please see the Supporting Information and methods for details of other wet lab experimental procedures.

### 2.6 Statistical Analysis

Bonferroni‐corrected significance levels with *p*‐value < 0.008 (0.05/6) for six lipid traits and *p*‐value < 0.005 (0.05/9) for nine drug targets were used to account for multiple testing adjustments accordingly. All data were shown as mean ± standard deviation (SD). Differences between groups were assessed by one‐way analysis of variance (ANOVA). Statistical analyses were performed using SPSS 20.0 software (SPSS Inc., Chicago, IL, USA) and R packages such as ‘TwoSampleMR’, ‘gtx’, ‘LDlinkR’, ‘MendelianRandomization’ and ‘coloc’ in R version 4.3. *p* values < 0.05 were considered statistically significant.

## 3 Results

### 3.1 Lipid Traits and Osteonecrosis Risk

**FIGURE 2:**
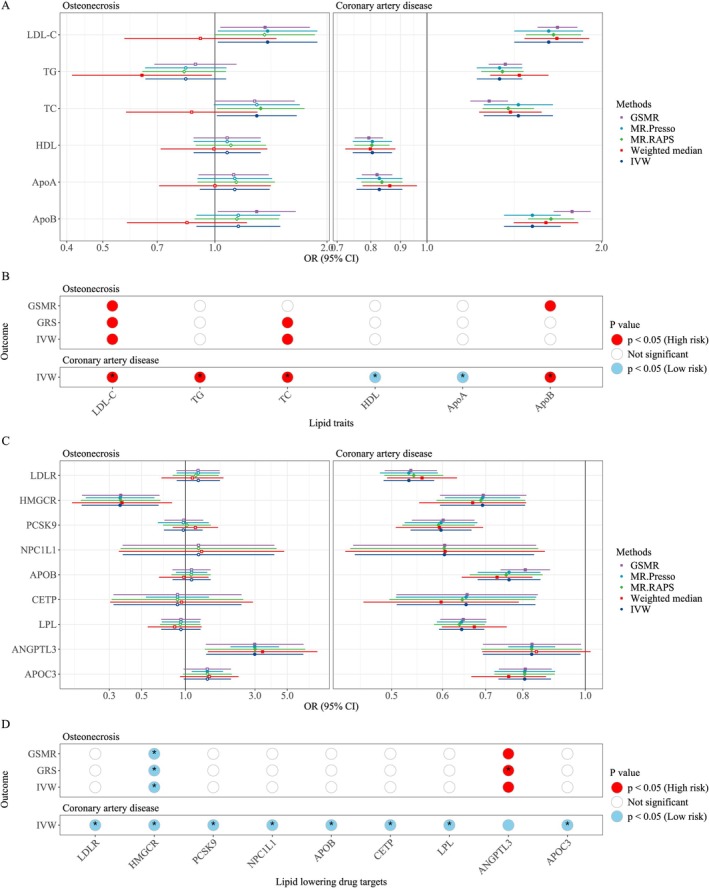
Mendelian randomisation analysis of lipid traits, lipid‐lowering drug targets and osteonecrosis risk. (A) Forest plot showed odds ratios for lipid traits associated with osteonecrosis and coronary artery disease (positive control). LDL‐C showed significant associations with osteonecrosis risk. All lipid traits were significantly associated with coronary artery disease. (B) Dot plot of IVW, GRS and GSMR analysis results. *p < 0.008 (0.05/6). (C) The forest plot depicts the odds ratios for various lipid‐lowering drug targets in relation to osteonecrosis and coronary artery disease (positive control). Drug targets HMGCR and ANGPTL3 showed significant associations with osteonecrosis risk. All assessed drug targets were significantly linked to coronary artery disease. (D) Dot plot of IVW, GRS and GSMR analysis results. *p < 0.005 (0.05/9)

After controlling for confounding factors such as smoking, systemic lupus erythematosus (SLE), alcohol consumption and diabetes that were associated with the osteonecrosis outcome, our results consistently revealed a positive relationship between LDL‐C and osteonecrosis (IVW: OR = 1.38 [95% CI, 1.02–1.88], *p* = 0.03; GSR: 1.001 [95% CI, 1.0002–1.002], *p* = 0.01; GSMR: 1.36 [95% CI, 1.03–1.80], *p* = 0.03) (Figure 2A,B). Interestingly, the causal association remained consistent even after adjusting for confounding factors in the multivariate MR analysis (Tables S3–S5 and Figure S2). All lipid traits showed positive results in the CAD positive control group, which supports the validity of our IVs and the credibility of our findings. The intercept term in MR‐Egger regression supported that there was no overall horizontal pleiotropy (Tables S6 and S7).

### 3.2 Lipid‐Lowering Drug Targets and Osteonecrosis Risk

We have identified a total of nine lipid‐lowering drug genes as the genetic instruments (Table S8). Our positive control analyses have confirmed significant associations between these genetically proxied drug targets and a reduced risk of CAD, which was consistent with previous research studies (Tables S9 and S10) [44, 45]. Genetically mimicking the HMGCR inhibitors was found to be significantly associated with a decreased risk of osteonecrosis in both the MR analysis (OR = 0.36 [95% CI 0.19–0.65], *p* = 8.5 × 10^−4^) and GRS analysis (OR = 0.994 [95% CI 0.992–0.997], *p* = 6.5 × 10^−4^). An interesting finding was noted for the genetic mimicry of ANGPTL3 inhibition on the harmful effect of osteonecrosis risk in the MR analysis (OR = 2.99 [95% CI 1.39–6.44], *p* = 5.2 × 10^−3^) and this observation was validated in GRS analysis (OR = 1.004 [95% CI 1.001–1.007], *p* = 0.004). The GSMR analysis results still confirmed the aforementioned findings. In contrast, other drug targets (APOC3, NPC1L1, PCSK9, APOB, CETP, LDLR and LPL) exhibited neutral effects on osteonecrosis outcomes based on their genetic resemblances (Figure 2C,D).

The absence of pleiotropy evidence in the MR‐Egger intercept enhanced the reliability of causal deductions (Tables S11 and S12). After accounting for LD correlation among genetic variants, the results remained consistent with the primary findings (Tables S13 and S14). Further investigation into more stringent LD thresholds did not significantly affect the width of confidence intervals or the stability of MR results for both HMGCR and ANGPTL3 in relation to osteonecrosis (Tables S15–S17).

### 3.3 Gene Expression and Osteonecrosis Risk

We utilised genetic variants associated with HMGCR expression in blood tissue from the eQTLGen database (Figure S3). Our findings indicate that an increase in blood tissue HMGCR expression was linked to a higher risk of osteonecrosis (IVW: OR = 1.37 [95% CI, 1.14–1.67]; *p* = 0.0009). Notably, when more stringent LD thresholds were applied, the results remained consistent (Table S18). Furthermore, ANGPTL3 expression was linked to a lower risk of osteonecrosis (IVW: OR = 0.76 [95% CI, 0.58–0.99]; *p* = 0.04) (Figure S4). However, the finding yielded minimal results when subjected to secondary analyses (Table S19).

To further explore this relationship, we also performed colocalisation analyses. HMGCR expression in blood tissue and osteonecrosis shared a causal variant (rs6453133) (blood tissue: PP.H4 = 0.999) (Table S20 and Figure S5), whereas the colocalisation finding of ANGPTL3 expression was poorly identified (liver tissue: PP.H4 = 0.012) (Table S21 and Figure S6).

### 3.4 Mediation Analysis: Mediation Role of Cytokines

**FIGURE 3:**
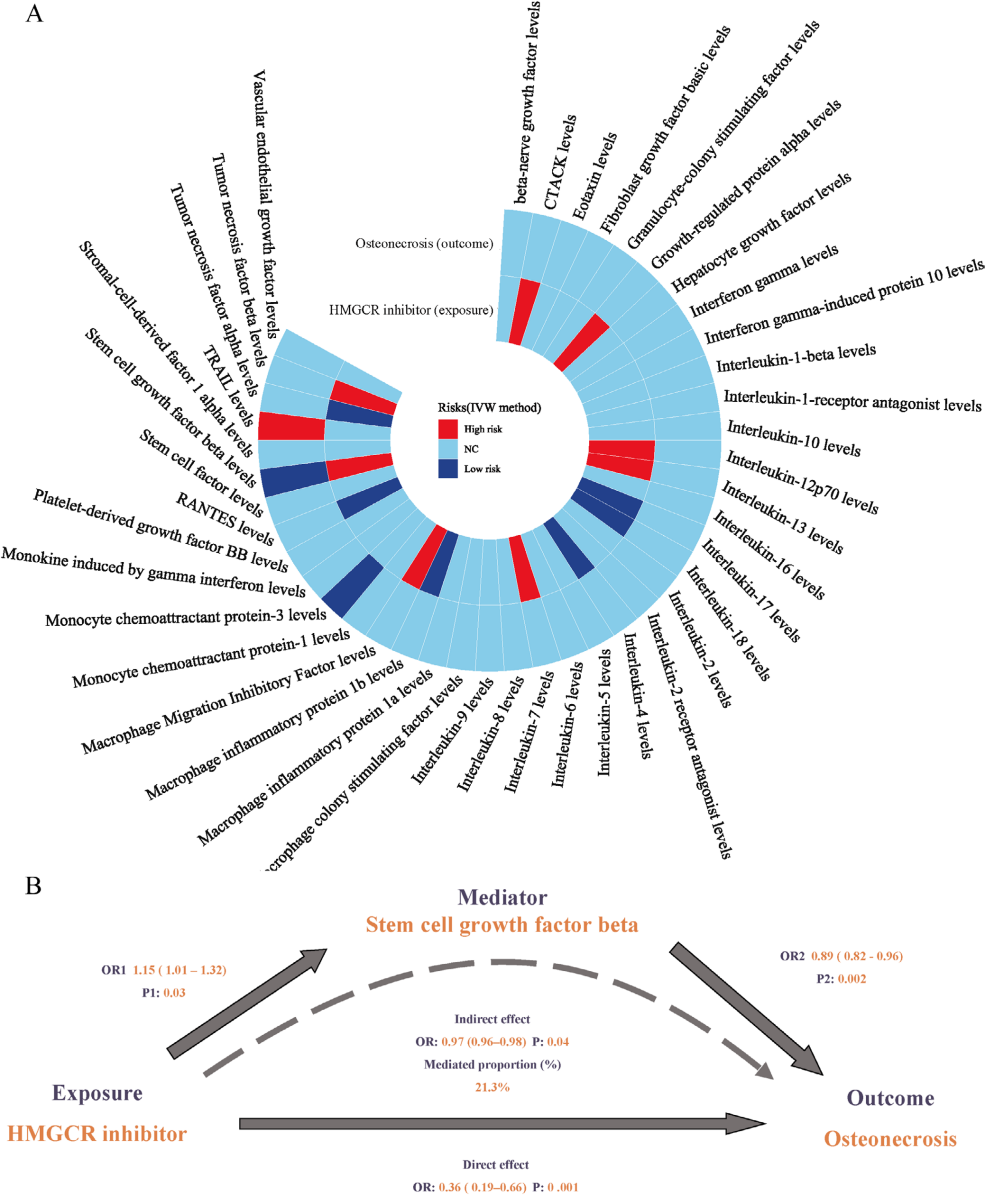
Mediation Mendelian randomisation analysis of HMGCR inhibitor's impact on osteonecrosis risk through cytokines. (A) The circle plot of association between HMGCR inhibitor, 41cytokines and the risk of osteonecrosis via mediation MR analysis. The inner circle represented the MR analysis between HMGCR inhibitor and 41 cytokines, while the outer circle represented the MR analysis between 41 cytokines and the risk of osteonecrosis. (B) Mediation analysis of the effect of HMGCR inhibitor on osteonecrosis via potential mediators under a two‐step Mendelian randomisation analysis framework. ‘Direct effect’ indicates the effect of HMGCR inhibitor on osteonecrosis risk after adjusting for the mediator (SCGF‐β). ‘Indirect effect’ indicates the effect of HMGCR inhibitor on osteonecrosis risk through the mediator (SCGF‐β).

As cytokines are potential risk factors for osteonecrosis and statins have anti‐inflammatory effects, cytokines may mediate the effects of HMGCR inhibitors (statins) on osteonecrosis. A two‐step MR analysis was conducted to investigate the mediating pathway from HMGCR inhibitor to osteonecrosis. The IVW results showed that HMGCR inhibitors were associated with 12 cytokines among the 41 tested, including cutaneous T cell‐attracting chemokine (CTACK) levels, macrophage migration inhibitory factor, tumour necrosis factor beta and alpha, SCGF‐β, macrophage inflammatory protein 1b, IL‐12p70, IL‐18, IL‐17, IL‐13, IL‐7, IL‐4 and growth‐regulated protein alpha levels. Some of these associations have already been studied [46]. In addition, we observed an association between osteonecrosis risk and tumour necrosis factor‐related apoptosis inducing ligand (TRAIL) and SCGF‐β among the 41 cytokines analysed (Figure 3A). Our analysis has revealed a clear causal relationship between the use of HMGCR inhibitors and a decreased risk of osteonecrosis, which occurs through the mediation of SCGF‐β (Tables S22 and S23). The impact of HMGCR inhibitor on osteonecrosis through SCGF‐β was found to be indirect with an OR of 0.98 (95% CI, 0.96–0.99; *p* = 0.04) (Figure 3B). Upon adjusting for SCGF‐β, the OR of HMGCR inhibitor on osteonecrosis increased from 0.35 (95% CI, 0.19–0.65; *p* = 8.5 × 10^−4^) to 0.36 (95% CI, 0.19–0.66; *p* = 0.001), indicating that a portion of the reduced risk of osteonecrosis associated with HMGCR inhibitor usage could be attributed to an elevated risk in SCGF‐β levels (mediation proportion: 21.3%) (Table S24). No heterogeneity was detected in our two‐step MR analysis.

### 3.5 Simvastatin Alleviated Steroid‐Induced Osteonecrosis of the Femoral Head (SONFH) Partially Mediated by SCGF‐β

**FIGURE 4:**
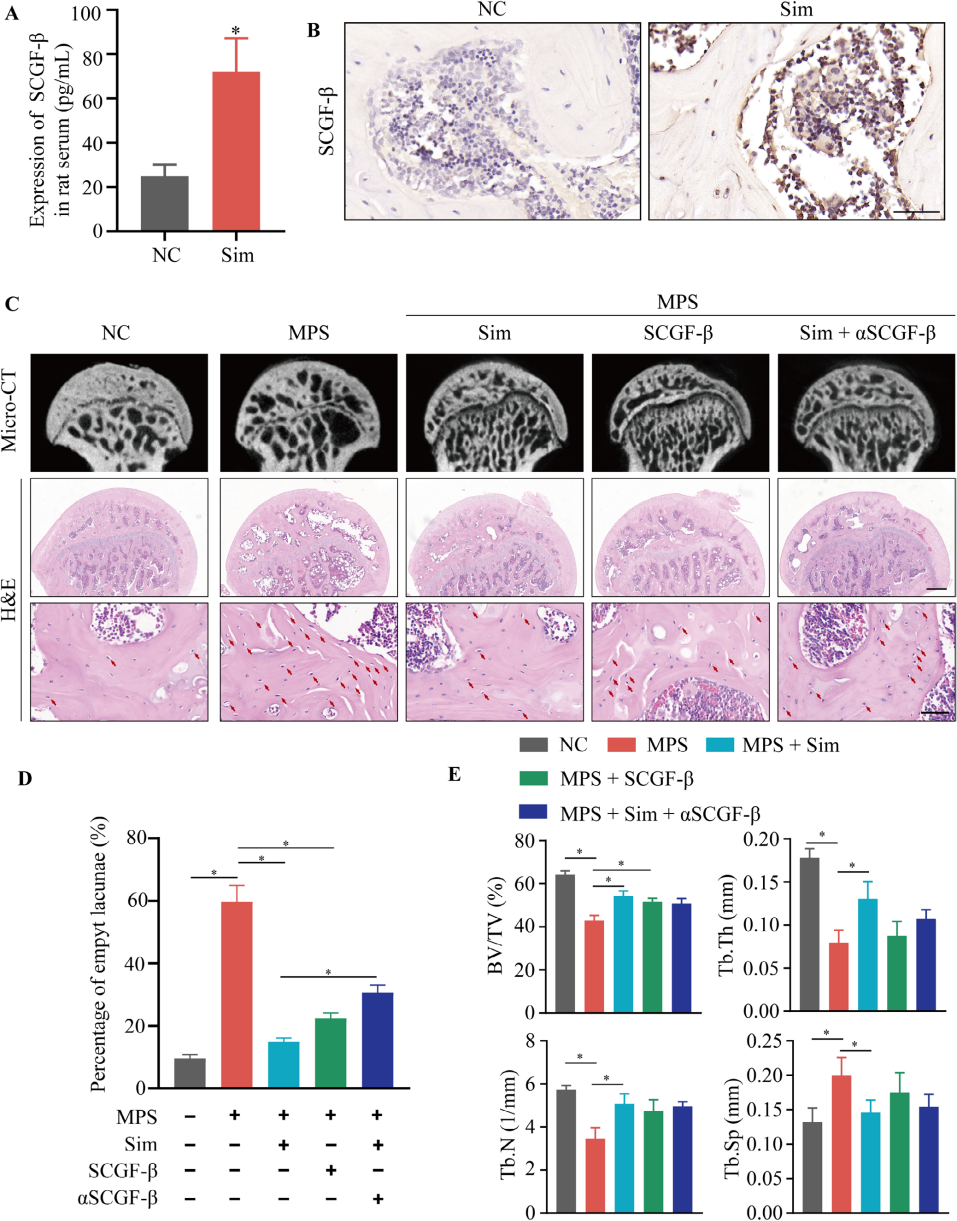
Simvastatin alleviated SONFH partially mediated by SCGF‐β. (A) The serum level of SCGF‐β in the simvastatin‐treated rat model. (B) IHC staining of SCGF‐β in the femoral heads of Simvastatin‐treated rat model. Scale bars: 50 μm. (C) Typical 3D‐μCT images and H&E staining images of femoral heads from SONFH rat models treated with the indicated drugs. The red arrow points to empty lacunae. Scale bars: 500 μm (for the middle images) and 50 μm (for the lower images). (D) Quantitative analysis of empty lacunae in the H&E staining images of rat femoral heads from SONFH and combined drug rat models. (E) Quantitative analysis of 3D‐μCT; BV/TV, bone volume per tissue volume; Tb.N, trabecular number; Tb.Sp, trabecular separation; Tb.Th, trabecular thickness. *p < 0.05. SCGF‐β, Purified SCGF‐β protein; Sim, simvastatin; αSCGF‐β, SCGF‐β neutralising antibody.

In order to determine whether simvastatin can regulate the levels of SCGF‐β in rat serum and femoral head, a simvastatin‐treated rat model was established. Following treatment with simvastatin, a significant increase in SCGF‐β level was found in rat serum (Figure 4A), and IHC staining indicated that treatment with simvastatin increased the expression of SCGF‐β in the intramedullary cells of the femoral head (Figure 4B). To assess whether simvastatin can inhibit SONFH in vivo and whether it acted partially through SCGF‐β, a rat model of SONFH was established, and oral simvastatin, femoral intramedullary injection of SCGF‐β purified protein, or a combination of oral simvastatin and intra‐medullary injection of SCGF‐β neutralising antibody were given. Three‐dimensional micro‐computed tomography (3D‐μCT) images (Figure 4C) and quantitative analyses (Figure 4D) of microstructural parameters such as bone volume to total volume (BV/TV), trabecular thickness (Tb.Th), trabecular separation (Tb.Sp) and trabecular number (Tb.N) revealed significant trabecular damage in the SONFH group. However, this damage was alleviated following treatment with either oral simvastatin or intra‐medullary injection of SCGF‐β recombinant protein. Moreover, intra‐medullary injection of SCGF‐β neutralising antibody inhibited the therapeutic effects of simvastatin in vivo. As depicted in Figure 4C,E, cystic degeneration and hyperplastic adipocytes were observed in the subchondral region of the femoral heads in the SONFH rats, with a significant reduction in trabecular thickness and numerous empty lacunae. However, treatment with either simvastatin or SCGF‐β recombinant protein markedly improved these pathological changes.

### 3.6 SCGF‐β Partly Mediated the Effect of Simvastatin on Promoting Osteogenesis and Inhibiting Adipogenesis of MSCs

**FIGURE 5:**
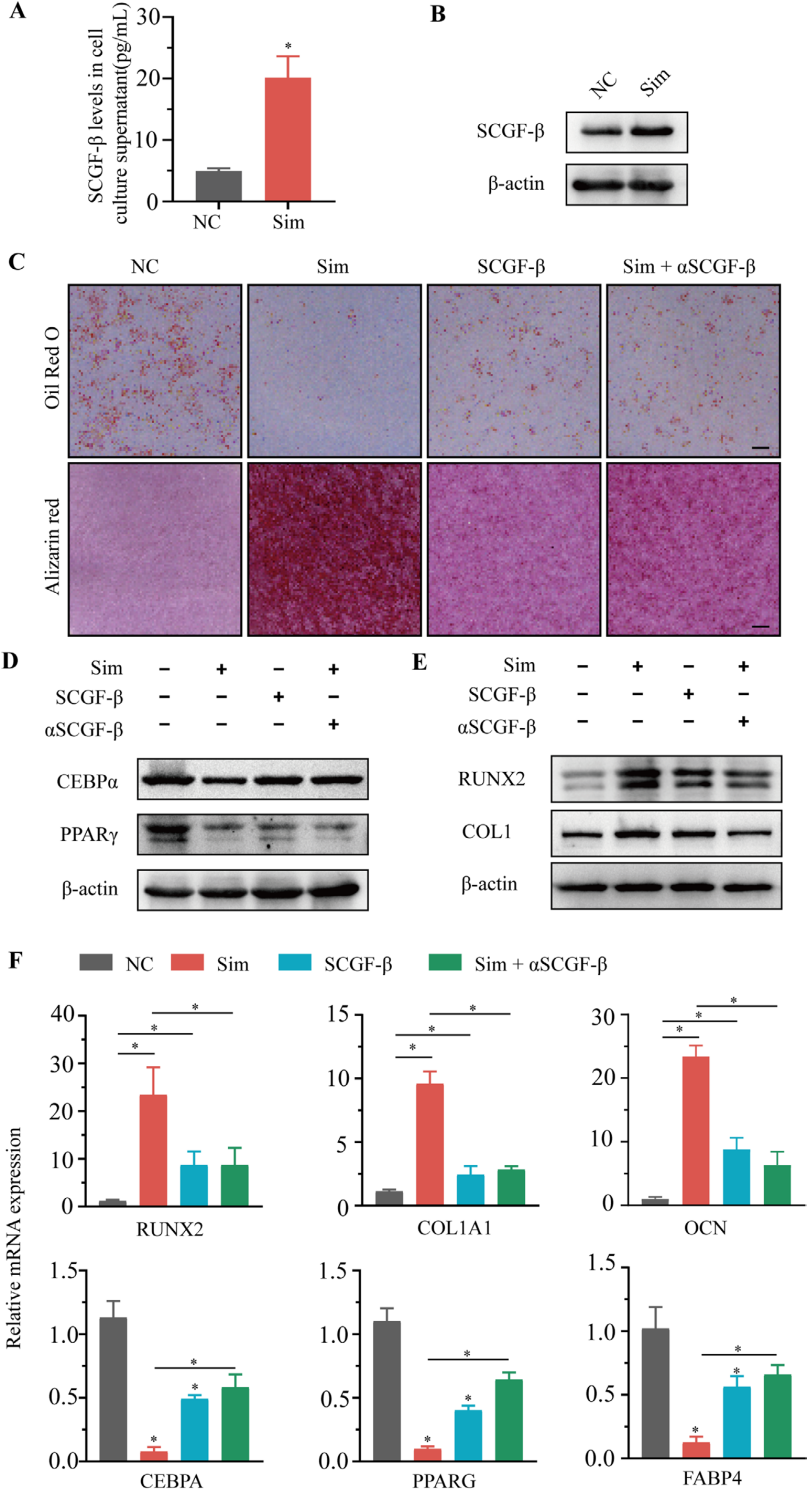
SCGF‐β partly mediated the effect of simvastatin on promoting osteogenesis and inhibiting adipogenesis of MSCs. (A) the MSCs were treated with simvastatin or vehicle, and the level of SCGF‐β in cell culture medium supernatant was detected by ELISA. (B) the MSCs were treated with simvastatin, and the expression level of SCGF‐β in cells was detected by WB. (C) the MSCs were treated with simvastatin, SCGF‐β recombinant protein, or simvastatin and SCGF‐β neutralising antibody, and then induced to adipogenesis or osteogenesis. After inducing adipogenesis for 8 days, oil‐red O staining was conducted. After inducing osteogenic differentiation for 14 days, alizarin red staining was conducted. Scale bars: 100 mm. (D) the MSCs were treated with simvastatin, SCGF‐β recombinant protein, or simvastatin and SCGF‐β neutralising antibody, and then induced to adipogenesis. After inducing adipogenesis for 8 days, the protein levels of C/EBPα and PPARγ were detected by WB. (E) the MSCs were treated with simvastatin, SCGF‐β recombinant protein, or simvastatin and SCGF‐β neutralising antibody, and then induced to osteogenic differentiation. After inducing osteogenic differentiation for 14 days, the protein levels of RUNX2 and COL1A1 were detected by WB. (F) the MSCs were treated with simvastatin, SCGF‐β recombinant protein, or simvastatin and SCGF‐β neutralising antibody, and then induced to adipogenesis or osteogenesis. After inducing adipogenesis for 8 days, the mRNA levels of C/EBPα, PPARγ and FABP4 were detected by RT‐qPCR. After inducing osteogenesis for 14 days, the mRNA levels of RUNX2, OCN and COL1A1 were detected by RT‐qPCR. *p < 0.05. SCGF‐β, Purified SCGF‐β Protein; Sim, simvastatin; αSCGF‐β, SCGF‐β neutralising antibody.

As shown in Figure 5A,B, treatment with simvastatin significantly increased the levels of SCGF‐β in both MSCs and the culture medium. As illustrated in Figure 5C–F, subsequent treatment with either simvastatin or SCGF‐β recombinant protein notably inhibited adipogenic differentiation of the MSCs, and meanwhile it enhanced the osteogenic capacity of MSCs. Interestingly, the use of SCGF‐β neutralising antibody counteracted the effects of simvastatin on the osteogenesis and adipogenesis of MSCs.

### 3.7 Wnt Pathway Mediated the Effects of Simvastatin–SCGF‐β Axis on the Adipogenesis and Osteogenesis of MSCs

**FIGURE 6:**
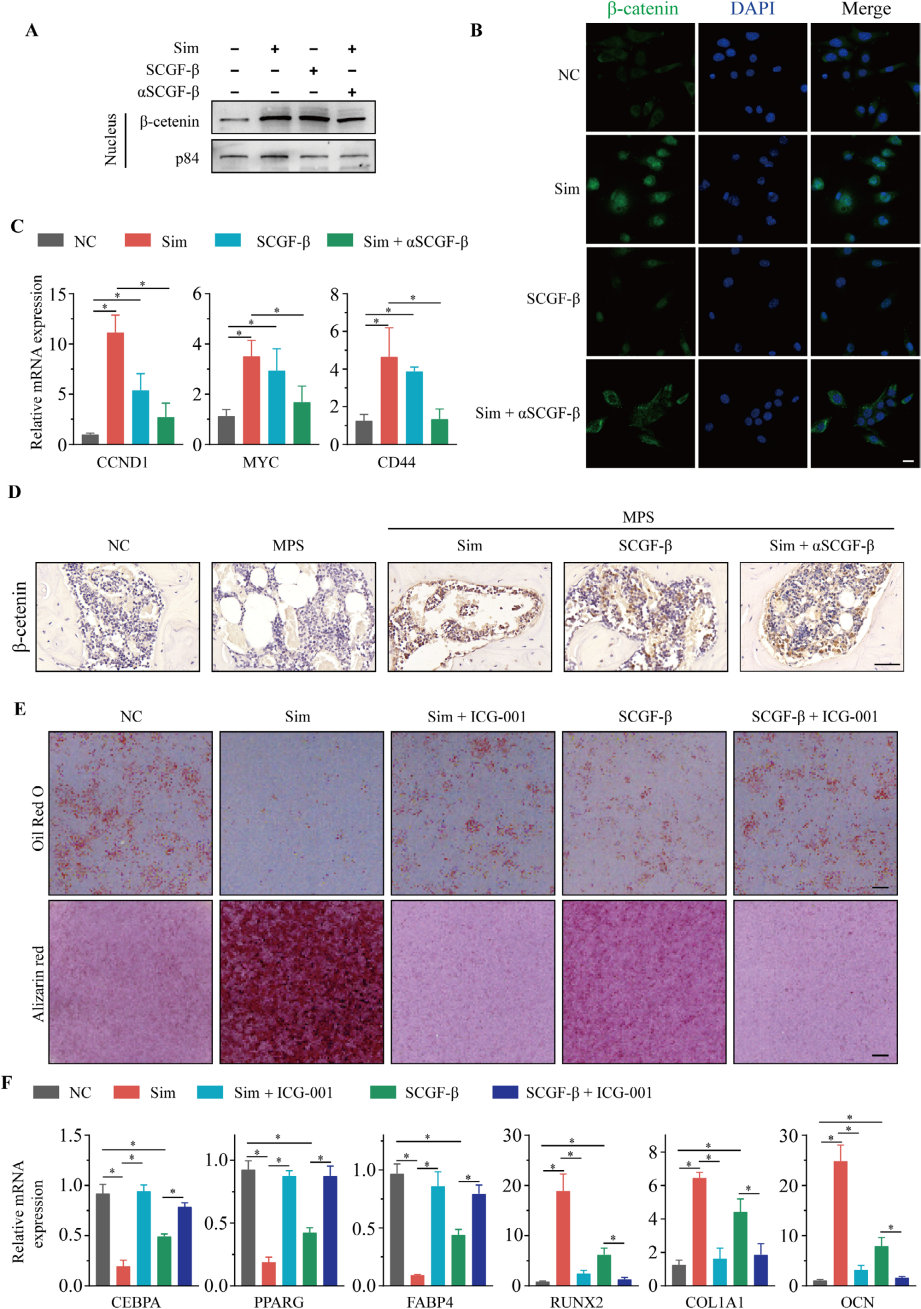
Wnt pathway mediated the effects of simvastatin‐SCGF‐β axis on the adipogenesis and osteogenesis of MSCs. (A) The MSCs were treated with simvastatin, SCGF‐β recombinant protein, or simvastatin and SCGF‐β neutralizing antibody, and the nuclear protein level of β‐catenin was detected. (B) the MSCs were treated with simvastatin, SCGF‐β recombinant protein, or simvastatin and SCGF‐β neutralising antibody, the expression and distribution of β‐catenin were detected by immunofluorescence staining. Scale bars: 10 μm. (C) the MSCs were treated with simvastatin, SCGF‐β recombinant protein, or simvastatin and SCGF‐beta neutralising antibody; the mRNA levels of CCND1, MYC and CD44 were detected by RT‐qPCR. (D) IHC was performed to detect the nuclear expressions of β‐catenin in femoral heads from SONFH rat models treated with the indicated drugs. Scale bars: 50 μm. (E, F) the MSCs were treated with simvastatin, SCGF‐β recombinant protein, SCGF‐β recombinant protein and ICG‐001, or simvastatin and ICG‐001, and then induced to adipogenesis or osteogenesis. Oil‐red O staining and alizarin red staining were conducted (E), and the mRNA levels of C/EBPα, PPARγ, FABP4, RUNX2, OCN and COL1A1 were detected by RT‐qPCR (F). Scale bars: 100 mm. *p < 0.05. SCGF‐β, purified SCGF‐β protein; Sim, simvastatin; αSCGF‐β, SCGF‐β neutralising antibody.

We observed that after treatment with either simvastatin or SCGF‐β recombinant protein, there was a significant increase in the nuclear β‐catenin levels in MSCs, as shown in Figure 6A,B. Concurrently, mRNA expression of typical downstream genes of the classical Wnt/β‐catenin signalling pathway, such as CCND1, MYC and CD44, also significantly increased, as depicted in Figure 6C. IHC staining of the femoral heads from SONFH rat models revealed that treatment with simvastatin or SCGF‐β promoted the nuclear expression of β‐catenin, while this effect was suppressed by the SCGF‐β neutralising antibody (Figure 6D). Subsequently, ICG‐001, a β‐catenin/TCF‐mediated transcription inhibitor, significantly reversed both the osteogenic promotion and adipogenic inhibition effects of simvastatin and SCGF‐β recombinant protein on MSCs (Figure 6E,F).

## 4 Discussion

In the present study, we identify lipid traits LDL‐C as causal risk factors of osteonecrosis. Through comprehensive evaluation of the effects of nine lipid‐lowering drugs, we have elucidated HMGCR as a promising therapeutic target for osteonecrosis treatment. Furthermore, statins (HMGCR inhibitors) are established to exert their anti‐osteonecrosis effects by regulating SCGF‐β expression. Through in vivo experiments, we found that oral administration of simvastatin or femoral intramedullary injection of purified SCGF‐β protein can effectively alleviate SONFH in the rat model. Furthermore, we found that simvastatin can stimulate the expression and secretion of SCGF‐β in MSCs, which subsequently inhibits adipogenesis and promotes osteogenesis of MSCs through activating Wnt signalling pathway.

In the MR analysis of 1385 osteonecrosis cases and 358,014 controls, LDL‐C was still associated with an increased risk of osteonecrosis by MVMR analysis, consistent with observational studies [7]. Interestingly, among the nine lipid‐lowering drug targets, HMGCR was the only drug target that significantly reduced the risk of osteonecrosis and passed colocalisation analysis validation. This finding was validated through various analytical methods using genetic instruments and a FinnGen osteonecrosis dataset. Our study provided strong evidence that HMGCR inhibitors, specifically statins, are promising drugs for osteonecrosis, from a different aspect compared to previous animal experiments [11, 13]. Suppressed osteogenic differentiation and increased bone marrow adipogenesis are critical pathological mechanisms underlying steroid‐ and alcohol‐induced ONFH. In this study, simvastatin exhibited a significant regulatory effect on promoting bone formation and inhibiting adipogenesis.

Considering that statins have anti‐inflammatory effects and can regulate various inflammatory cytokines [47], we conducted mediation MR analyses for 41 cytokines. Although we found that statins were associated with 12 cytokines, only SCGF‐β of these factors was associated with osteonecrosis. However, we found that simvastatin effectively increased the expression level of SCGF‐β both in vitro and in vivo. Stem Cell Growth Factor (SCGF) is a secreted protein encoded by the gene CLEC11A, belonging to the C‐type lectin superfamily. SCGF protein exists in two different forms, SCGF‐α (Osteolectin) as the full‐length form (35 kDa), and SCGF‐β as the truncated form (27 kDa), lacking 78 amino acids in its C‐terminal lectin domain. SCGF‐α secreted by MSCs has been confirmed to be able to act on its own integrin α11 receptor to promote Wnt pathway activation and osteogenic differentiation [48]. Similarly, our research showed that SCGF‐β could also promote osteogenesis and inhibit adipogenesis via activating Wnt signalling. And SCGF‐β partially mediated the anti‐osteonecrosis effect of statins through this newly discovered mechanism.

In this study, no evidence was found to support the beneficial effects of other lipid‐lowering medications on osteonecrosis. Conversely, we found that genetic mimicry of ANGPTL3 inhibition increases the risk of osteonecrosis. This may be because ANGPTL3 itself has a vital role in promoting angiogenesis [49, 50], and inhibiting ANGPTL3 may lead to impaired angiogenesis and worsening of osteonecrosis, which needs to be further studied.

In explaining the results of this study, some limitations should be considered. Firstly, due to limited data on osteonecrosis, we could not perform secondary analyses to validate the stability of the results. Furthermore, since our study only included individuals of European ancestry, these findings may not necessarily apply to other ethnic groups.

We believe that both statins and SCGF‐β hold tremendous potential in the field of osteonecrosis treatment. Further clinical research is strongly necessary to confirm the safety and efficacy of statins and SCGF‐β in the treatment of osteonecrosis. Additionally, the detailed molecular mechanisms underlying the effect of statins and SCGF‐β need to be elucidated, which will provide more insights and possibilities for developing new treatment strategies.

## 5 Conclusions

In this study, utilising Mendelian randomisation methods, we demonstrated that lipid traits have a causal relationship with osteonecrosis risk. Among the nine lipid‐lowering drug targets, HMGCR is a promising therapeutic target for osteonecrosis treatment, and statin may exert anti‐osteonecrotic effects through SCGF‐β. Finally, we validated these findings through in vivo and in vitro experiments, demonstrating that simvastatin promotes osteogenesis and inhibits adipogenesis of MSCs, thereby halting the progression of osteonecrosis, with SCGF‐β potentially playing a role in this process. This study provides a novel perspective on the relationship between lipid metabolism and osteonecrosis, as well as a potential effective therapeutic treatment for osteonecrosis.

## Author Contributions

**Fangzhou Fan:** writing – review and editing, writing – original draft, visualisation, software, methodology, formal analysis, conceptualisation. **Yu Chen:** writing – review and editing, writing – original draft, visualisation, investigation, methodology, conceptualisation. **Weiyan Peng:** visualisation, investigation. **Wenlong Yan:** methodology, project administration, funding acquisition. **Hao Tan:** methodology. **Chengxuan Zhang:** methodology. **Siyu Tan:** investigation. **Qian Xiao:** resources, project administration, funding acquisition. **Yuan Gao:** resources, project administration. **Jian Zhang:** writing – review and editing, funding acquisition. **Liu Lei:** resources, writing – review and editing, supervision, methodology, conceptualisation. **Chengjie Lian:** resources, writing – review and editing, supervision, methodology, conceptualisation. All authors read and approved the final version of the manuscript.

## Funding

This work was supported by the Natural Science Foundation of China (No. 82473020, 82102610, 32271179); Fujian Provincial Natural Science Foundation of China (No. 2025J01127); Chongqing Natural Science Foundation (No. CSTB2023NSCQ‐LZX0018; CSTB2022NSCQ‐MSX0929; CSTB2024NSCQ‐MSX0952); Chongqing medical scientific research project—Joint project of Chongqing Health Commission and Science and Technology Bureau (No. 2024GDRC006); The Science and Technology Research Project of Chongqing Education Commission (No. KJQN202200404, KJQN202200441); the Science and Technology Research Project of Chongqing Sports Bureau (No. C202124, A202206); Research project of Fujian Medical University Union Hospital (No. 2024XH034); the Doctoral Research Innovation Project of the First Affiliated Hospital of Chongqing Medical University (No. CYYY‐BSYJSKYCXXM202404) and the Graduate Advisor Team Project of the First Affiliated Hospital of Chongqing Medical University (No. CYYY‐DSTDXM‐202407, CYYY‐XKDFJH‐DSTD‐202405).

## Ethics Statement

The authors have nothing to report.

## Consent

The authors have nothing to report.

## Conflicts of Interest

The authors declare no conflicts of interest.

## Supporting information


**Figures S1–S6:** jcmm70967‐sup‐0001‐FiguresS1‐S6.docx.

**Data S1:** jcmm70967‐sup‐0002‐DataS1.docx.

**Tables S1–S24:** jcmm70967‐sup‐0003‐TablesS1‐S24.xlsx.

## Data Availability

See the Supporting Information and methods for details of other wet lab experimental procedures. All data are publicly available. Detailed information for these datasets is summarised in Table S2. The source codes for computing work are available at https://github.com/fangzhoufan/statins_osteonecrosis.

## References

[1] M. A. Mont , H. S. Salem , N. S. Piuzzi , S. B. Goodman , and L. C. Jones , “Nontraumatic Osteonecrosis of the Femoral Head: Where Do We Stand Today?: A 5‐Year Update,” Journal of Bone and Joint Surgery. American Volume 102, no. 12 (2020): 1084–1099. 32282421 10.2106/JBJS.19.01271 PMC7508290

[2] D. W. Zhao , M. Yu , K. Hu , et al., “Prevalence of Nontraumatic Osteonecrosis of the Femoral Head and Its Associated Risk Factors in the Chinese Population: Results From a Nationally Representative Survey,” Chinese Medical Journal 128, no. 21 (2015): 2843–2850. 26521779 10.4103/0366-6999.168017 PMC4756878

[3] M. A. Mont and D. S. Hungerford , “Non‐Traumatic Avascular Necrosis of the Femoral Head,” Journal of Bone and Joint Surgery. American Volume 77, no. 3 (1995): 459–474. 7890797 10.2106/00004623-199503000-00018

[4] D. Zhao , F. Zhang , B. Wang , et al., “Guidelines for Clinical Diagnosis and Treatment of Osteonecrosis of the Femoral Head in Adults (2019 Version),” Journal of Orthopaedic Translation 21 (2020): 100–110. 32309135 10.1016/j.jot.2019.12.004 PMC7152793

[5] C. G. Zalavras and J. R. Lieberman , “Osteonecrosis of the Femoral Head: Evaluation and Treatment,” Journal of the American Academy of Orthopaedic Surgeons 22, no. 7 (2014): 455–464. 24966252 10.5435/JAAOS-22-07-455

[6] A. Postiglione and C. Napoli , “Hyperlipidaemia and Atherosclerotic Cerebrovascular Disease,” Current Opinion in Lipidology 6, no. 4 (1995): 236–242. 7670753 10.1097/00041433-199508000-00008

[7] Y. Song , Z. Du , B. Chen , et al., “Association of SREBP2 Gene Polymorphisms With the Risk of Osteonecrosis of the Femoral Head Relates to Gene Expression and Lipid Metabolism Disorders,” Molecular Medicine Reports 16, no. 5 (2017): 7145–7153. 28901487 10.3892/mmr.2017.7473

[8] T. Kabata , T. Kubo , T. Matsumoto , et al., “Onset of Steroid‐Induced Osteonecrosis in Rabbits and Its Relationship to Hyperlipaemia and Increased Free Fatty Acids,” Rheumatology 44, no. 10 (2005): 1233–1237. 15972352 10.1093/rheumatology/keh721

[9] Q. Xu , H. Chen , S. Chen , et al., “Development and Validation of a Nomogram for Predicting the Probability of Nontraumatic Osteonecrosis of the Femoral Head in the Chinese Population,” Scientific Reports 10, no. 1 (2020): 20660. 33244062 10.1038/s41598-020-77693-9 PMC7691506

[10] X. Yu , S. Zhang , B. Zhang , and M. Dai , “Relationship of Idiopathic Femoral Head Necrosis With Blood Lipid Metabolism and Coagulation Function: A Propensity Score‐Based Analysis,” Frontiers in Surgery 9 (2022): 938565. 36684312 10.3389/fsurg.2022.938565 PMC9852306

[11] K. Pengde , P. Fuxing , S. Bin , Y. Jing , and C. Jingqiu , “Lovastatin Inhibits Adipogenesis and Prevents Osteonecrosis in Steroid‐Treated Rabbits,” Joint Bone Spine 75, no. 6 (2008): 696–701. 18620886 10.1016/j.jbspin.2007.12.008

[12] Y. Nozaki , K. Kumagai , N. Miyata , and M. Niwa , “Pravastatin Reduces Steroid‐Induced Osteonecrosis of the Femoral Head in Shrsp Rats,” Acta Orthopaedica 83, no. 1 (2012): 87–92. 22313369 10.3109/17453674.2011.641103 PMC3278663

[13] Y. Jiang , Y. Zhang , H. Zhang , et al., “Pravastatin Prevents Steroid‐Induced Osteonecrosis in Rats by Suppressing Ppargamma Expression and Activating Wnt Signalling Pathway,” Experimental Biology and Medicine 239, no. 3 (2014): 347–355. 24510055 10.1177/1535370213519215

[14] K. Sakamoto , M. Osaki , A. Hozumi , et al., “Simvastatin Suppresses Dexamethasone‐Induced Secretion of Plasminogen Activator Inhibitor‐1 in Human Bone Marrow Adipocytes,” BMC Musculoskeletal Disorders 12, no. 1 (2011): 82. 21524281 10.1186/1471-2474-12-82 PMC3114799

[15] J. W. Pritchett , “Statin Therapy Decreases the Risk of Osteonecrosis in Patients Receiving Steroids,” Clinical Orthopaedics and Related Research 386 (2001): 173–178. 10.1097/00003086-200105000-00022 11347831

[16] M. Ajmal , A. J. Matas , M. Kuskowski , and E. Y. Cheng , “Does Statin Usage Reduce the Risk of Corticosteroid‐Related Osteonecrosis in the Renal Transplant Population?,” Orthopedic Clinics of North America 40, no. 2 (2009): 235–239. 19358908 10.1016/j.ocl.2009.01.004 PMC2801433

[17] E. R. Finch , M. A. Payton , D. A. Jenkins , et al., “Fenofibrate Reduces Osteonecrosis Without Affecting Antileukemic Efficacy in Dexamethasone‐Treated Mice,” Haematologica 106, no. 8 (2021): 2095–2101. 32675219 10.3324/haematol.2020.252767 PMC8327737

[18] K. Nguyen and B. D. Mitchell , “A Guide to Understanding Mendelian Randomization Studies,” Arthritis Care and Research 76, no. 11 (2024): 1451–1460. 39030941 10.1002/acr.25400 PMC11833605

[19] C. A. Emdin , A. V. Khera , and S. Kathiresan , “Mendelian Randomization,” JAMA 318, no. 19 (2017): 1925–1926. 29164242 10.1001/jama.2017.17219

[20] N. M. Davies , M. V. Holmes , and S. G. Davey , “Reading Mendelian Randomization Studies: A Guide, Glossary, and Checklist for Clinicians,” BMJ (Clinical Research ed.) 362 (2018): k601. 10.1136/bmj.k601 PMC6041728 30002074

[21] D. Gill , M. K. Georgakis , F. Koskeridis , et al., “Use of Genetic Variants Related to Antihypertensive Drugs to Inform on Efficacy and Side Effects,” Circulation 140, no. 4 (2019): 270–279. 31234639 10.1161/CIRCULATIONAHA.118.038814 PMC6687408

[22] W. R. Reay and M. J. Cairns , “Advancing the Use of Genome‐Wide Association Studies for Drug Repurposing,” Nature Reviews. Genetics 22, no. 10 (2021): 658–671. 10.1038/s41576-021-00387-z 34302145

[23] M. S. Sabatine , R. P. Giugliano , A. C. Keech , et al., “Evolocumab and Clinical Outcomes in Patients With Cardiovascular Disease,” New England Journal of Medicine 376, no. 18 (2017): 1713–1722. 28304224 10.1056/NEJMoa1615664

[24] Z. Ren and L. Zhou , “Association of Statin Use With Osteoporosis Risk: A Drug‐Targeted Mendelian Randomization Study,” Inflammopharmacology 32, no. 2 (2024): 1253–1261. 38363475 10.1007/s10787-024-01441-y

[25] X. Chen , X. Huang , Y. Liu , Z. Zhang , and J. Chen , “Assessing the Causal Associations of Different Types of Statin Use and Knee/Hip Osteoarthritis: A Mendelian Randomization Study,” PLoS One 19, no. 4 (2024): e297766. 10.1371/journal.pone.0297766 PMC11034643 38648228

[26] C. J. Willer , E. M. Schmidt , S. Sengupta , et al., “Discovery and Refinement of Loci Associated With Lipid Levels,” Nature Genetics 45, no. 11 (2013): 1274–1283. 24097068 10.1038/ng.2797 PMC3838666

[27] A. R. Barton , M. A. Sherman , R. E. Mukamel , and P. R. Loh , “Whole‐Exome Imputation Within UK Biobank Powers Rare Coding Variant Association and Fine‐Mapping Analyses,” Nature Genetics 53, no. 8 (2021): 1260–1269. 34226706 10.1038/s41588-021-00892-1 PMC8349845

[28] D. M. Lloyd‐Jones , P. B. Morris , C. M. Ballantyne , et al., “2022 ACC Expert Consensus Decision Pathway on the Role of Nonstatin Therapies for LDL‐Cholesterol Lowering in the Management of Atherosclerotic Cardiovascular Disease Risk: A Report of the American College of Cardiology Solution Set Oversight Committee,” Journal of the American College of Cardiology 80, no. 14 (2022): 1366–1418. 36031461 10.1016/j.jacc.2022.07.006

[29] F. Mach , C. Baigent , A. L. Catapano , et al., “2019 ESC/EAS Guidelines for the Management of Dyslipidaemias: Lipid Modification to Reduce Cardiovascular Risk,” European Heart Journal 41, no. 1 (2020): 111–188. 31504418 10.1093/eurheartj/ehz455

[30] J. Boren , M. R. Taskinen , E. Bjornson , and C. J. Packard , “Metabolism of Triglyceride‐Rich Lipoproteins in Health and Dyslipidaemia,” Nature Reviews. Cardiology 19, no. 9 (2022): 577–592. 35318466 10.1038/s41569-022-00676-y

[31] P. M. Ridker , “Ldl Cholesterol: Controversies and Future Therapeutic Directions,” Lancet 384, no. 9943 (2014): 607–617. 25131980 10.1016/S0140-6736(14)61009-6

[32] S. Ross , M. D'Mello , S. S. Anand , et al., “Effect of Bile Acid Sequestrants on the Risk of Cardiovascular Events: A Mendelian Randomization Analysis,” Circulation. Cardiovascular Genetics 8, no. 4 (2015): 618–627. 26043746 10.1161/CIRCGENETICS.114.000952

[33] “The GTEx Consortium Atlas of Genetic Regulatory Effects Across Human Tissues,” Science 369, no. 6509 (2020): 1318–1330. 32913098 10.1126/science.aaz1776 PMC7737656

[34] U. Vosa , A. Claringbould , H. J. Westra , et al., “Large‐Scale Cis‐ and Trans‐eQTL Analyses Identify Thousands of Genetic Loci and Polygenic Scores That Regulate Blood Gene Expression,” Nature Genetics 53, no. 9 (2021): 1300–1310. 34475573 10.1038/s41588-021-00913-z PMC8432599

[35] M. I. Kurki , J. Karjalainen , P. Palta , et al., “FinnGen Provides Genetic Insights From a Well‐Phenotyped Isolated Population,” Nature 613, no. 7944 (2023): 508–518. 36653562 10.1038/s41586-022-05473-8 PMC9849126

[36] A. V. Ahola‐Olli , P. Wurtz , A. S. Havulinna , et al., “Genome‐Wide Association Study Identifies 27 Loci Influencing Concentrations of Circulating Cytokines and Growth Factors,” American Journal of Human Genetics 100, no. 1 (2017): 40–50. 27989323 10.1016/j.ajhg.2016.11.007 PMC5223028

[37] M. Nikpay , A. Goel , H. H. Won , et al., “A Comprehensive 1000 Genomes‐Based Genome‐Wide Association Meta‐Analysis of Coronary Artery Disease,” Nature Genetics 47, no. 10 (2015): 1121–1130. 26343387 10.1038/ng.3396 PMC4589895

[38] C. Giambartolomei , D. Vukcevic , E. E. Schadt , et al., “Bayesian Test for Colocalisation Between Pairs of Genetic Association Studies Using Summary Statistics,” PLoS Genetics 10, no. 5 (2014): e1004383. 24830394 10.1371/journal.pgen.1004383 PMC4022491

[39] R. Ding , X. Zou , Y. Qin , et al., “xQTLbiolinks: A Comprehensive and Scalable Tool for Integrative Analysis of Molecular Qtls,” Briefings in Bioinformatics 25, no. 1 (2023): bbad440. 38058186 10.1093/bib/bbad440 PMC10701093

[40] M. Lau , C. Wigmann , S. Kress , T. Schikowski , and H. Schwender , “Evaluation of Tree‐Based Statistical Learning Methods for Constructing Genetic Risk Scores,” BMC Bioinformatics 23, no. 1 (2022): 97. 35313824 10.1186/s12859-022-04634-w PMC8935722

[41] F. Zhou , G. Zhang , Y. Wu , and Y. Xiong , “Inflammasome Complexes: Crucial Mediators in Osteoimmunology and Bone Diseases,” International Immunopharmacology 110 (2022): 109072. 35978515 10.1016/j.intimp.2022.109072

[42] B. Woolf , L. Zagkos , and D. Gill , “TwoStepCisMR: A Novel Method and R Package for Attenuating Bias in Cis‐Mendelian Randomization Analyses,” Genes (Basel) 13, no. 9 (2022): 1541. 36140709 10.3390/genes13091541 PMC9498486

[43] Y. Chen , B. Tang , W. Jiang , et al., “miR‐486‐5p Attenuates Steroid‐Induced Adipogenesis and Osteonecrosis of the Femoral Head via TBX2/P21 Axis,” Stem Cells 41, no. 7 (2023): 711–723. 37210668 10.1093/stmcls/sxad038

[44] T. G. Richardson , G. M. Leyden , Q. Wang , et al., “Characterising Metabolomic Signatures of Lipid‐Modifying Therapies Through Drug Target Mendelian Randomization,” PLoS Biology 20, no. 2 (2022): e3001547. 35213538 10.1371/journal.pbio.3001547 PMC8906647

[45] Q. Wang , C. Oliver‐Williams , O. T. Raitakari , et al., “Metabolic Profiling of Angiopoietin‐Like Protein 3 and 4 Inhibition: A Drug‐Target Mendelian Randomization Analysis,” European Heart Journal 42, no. 12 (2021): 1160–1169. 33351885 10.1093/eurheartj/ehaa972 PMC7982288

[46] K. Zhang , W. Liu , and H. Liang , “Effect of Statins on Sepsis and Inflammatory Factors: A Mendelian Randomization Study,” European Journal of Clinical Investigation 54, no. 5 (2024): e14164. 38229409 10.1111/eci.14164

[47] L. M. Biasucci , G. Biasillo , and A. Stefanelli , “Inflammatory Markers, Cholesterol and Statins: Pathophysiological Role and Clinical Importance,” Clinical Chemistry and Laboratory Medicine 48, no. 12 (2010): 1685–1691. 20868311 10.1515/CCLM.2010.277

[48] B. Shen , K. Vardy , P. Hughes , et al., “Integrin Alpha11 Is an Osteolectin Receptor and Is Required for the Maintenance of Adult Skeletal Bone Mass,” eLife 8 (2019): e42274. 30632962 10.7554/eLife.42274 PMC6349404

[49] F. Luo , P. Wu , J. Chen , et al., “ANGPTL3 Possibly Promotes Cardiac Angiogenesis Through Improving the Proangiogenic Ability of Endothelial Progenitor Cells After Myocardial Infarction,” Lipids in Health and Disease 17, no. 1 (2018): 184. 30086775 10.1186/s12944-018-0835-0 PMC6081830

[50] S. Jiang , G. H. Qiu , N. Zhu , Z. Y. Hu , D. F. Liao , and L. Qin , “ANGPTL3: A Novel Biomarker and Promising Therapeutic Target,” Journal of Drug Targeting 27, no. 8 (2019): 876–884. 30615486 10.1080/1061186X.2019.1566342

