# Supplementary material for: Statins Regulate Stem Cell Growth Factor‐β to Balance Osteogenesis and Adipogenesis in Mesenchymal Stem Cells, Endowing Anti‐Osteonecrosis Effects
Source: J Cell Mol Med. 2025 Nov 26;29(22):e70967. doi: 10.1111/jcmm.70967 (PMC12648295; doi:10.1111/jcmm.70967)
Supplement: Supplementary file 1 — Figures S1–S6: jcmm70967‐sup‐0001‐FiguresS1‐S6.docx. [file JCMM-29-e70967-s001.docx]

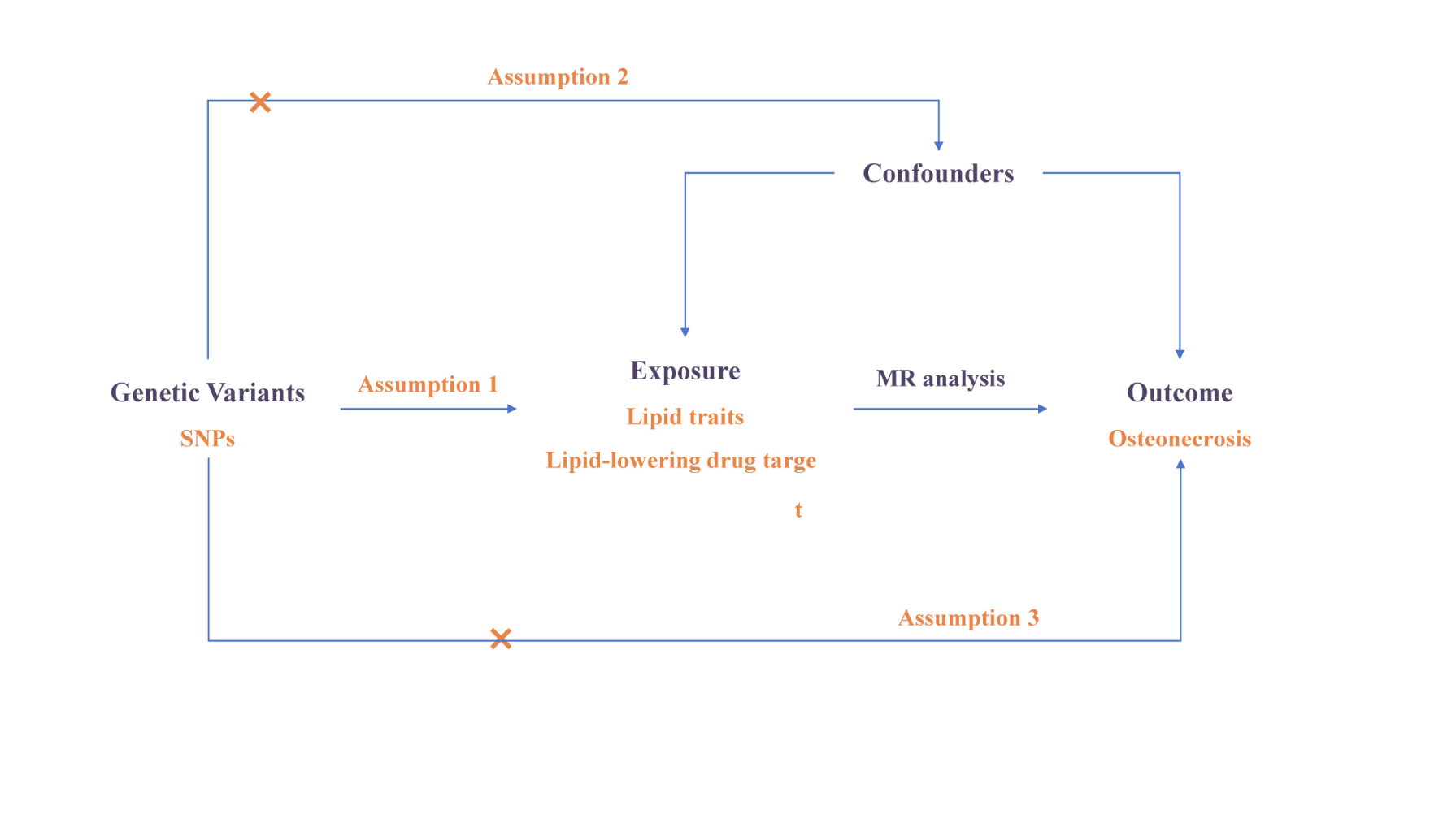


**Figure S1. Assumptions of the Mendelian randomization study design.**

Three fundamental assumptions were behind the MR approach: (1) strong association between the IVs and exposure (“relevance”); (2) absence of any associations between IVs and confounding factors that may influence both exposure and outcome (“independence”); (3) exclusive influence of IVs on outcome through their impact on exposure (“exclusion restriction”).

**
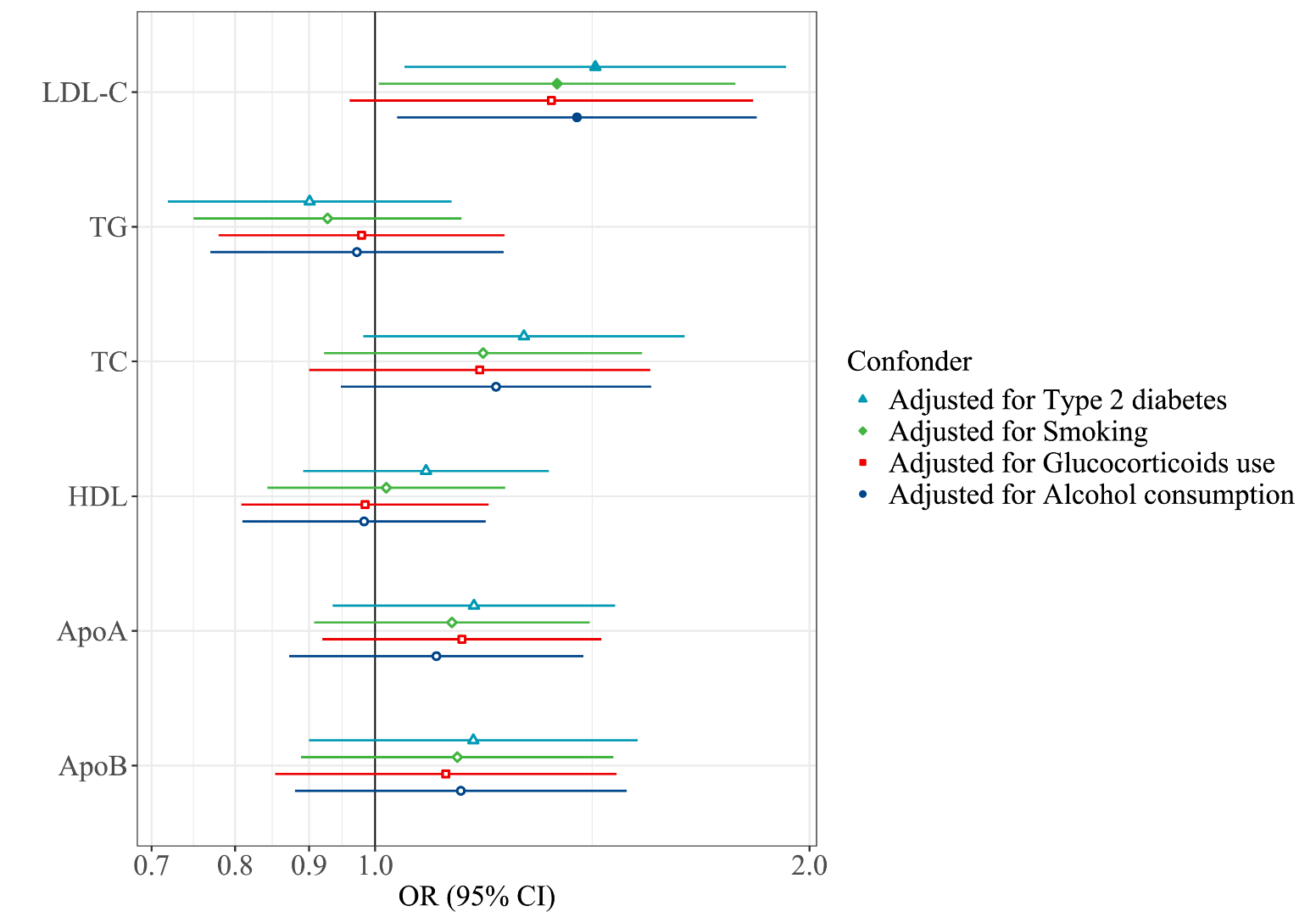
**

**Figure S2. Association between genetically proxied lipid traits and osteonecrosis after adjustment for potential mediators.**

**
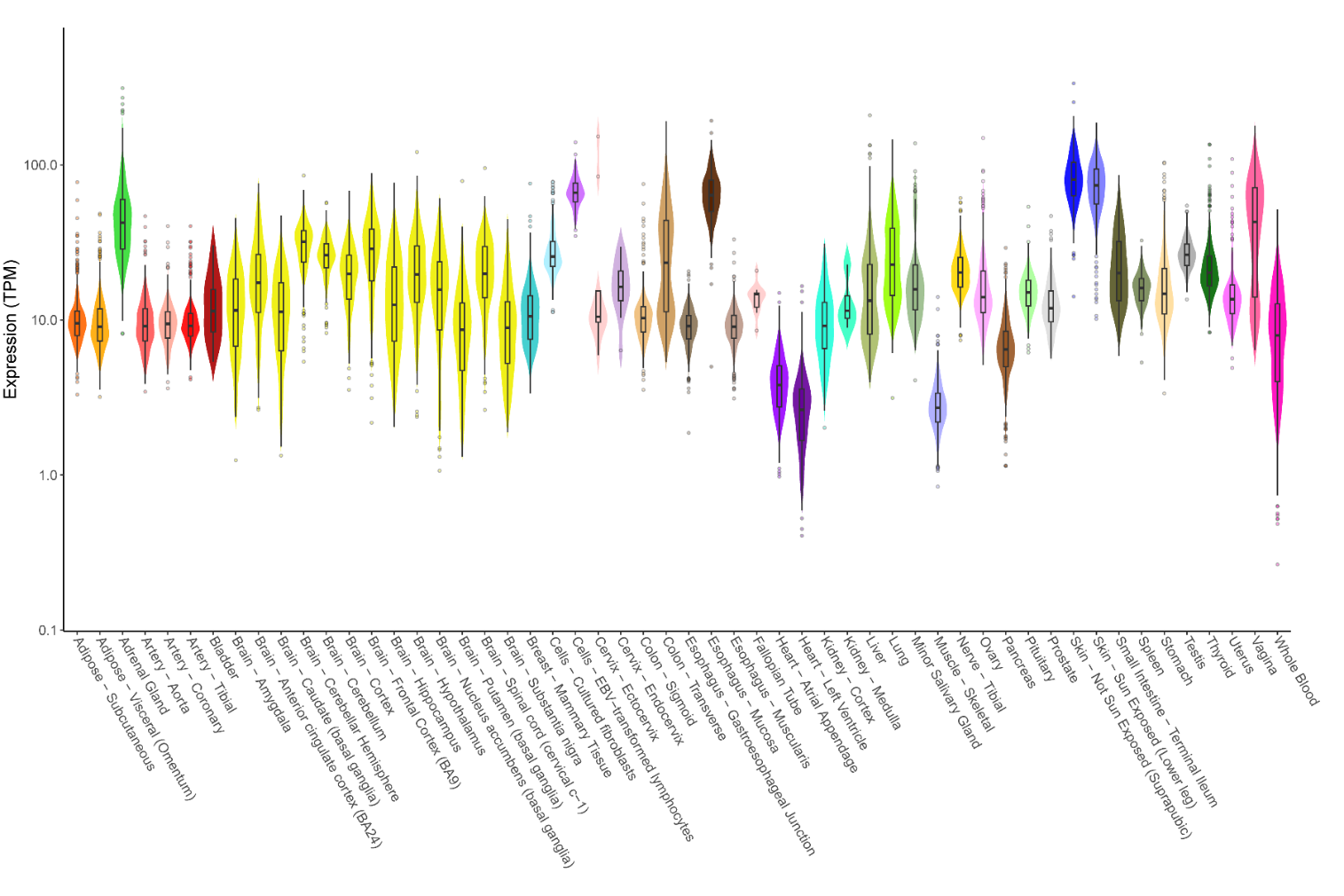
**

**Figure S3. Relative HMGCR mRNA expression in 49 tissues in GTEx-V8 datasets**

The figure represents the relative mRNA expression levels of the HMGCR gene normalized across 49 different human tissues from the GTEx-V8 datasets.

Abbreviations: TPM, transcripts per million; HMGCR, 3-hydroxy-3-methylglutaryl coenzyme A reductase.

**
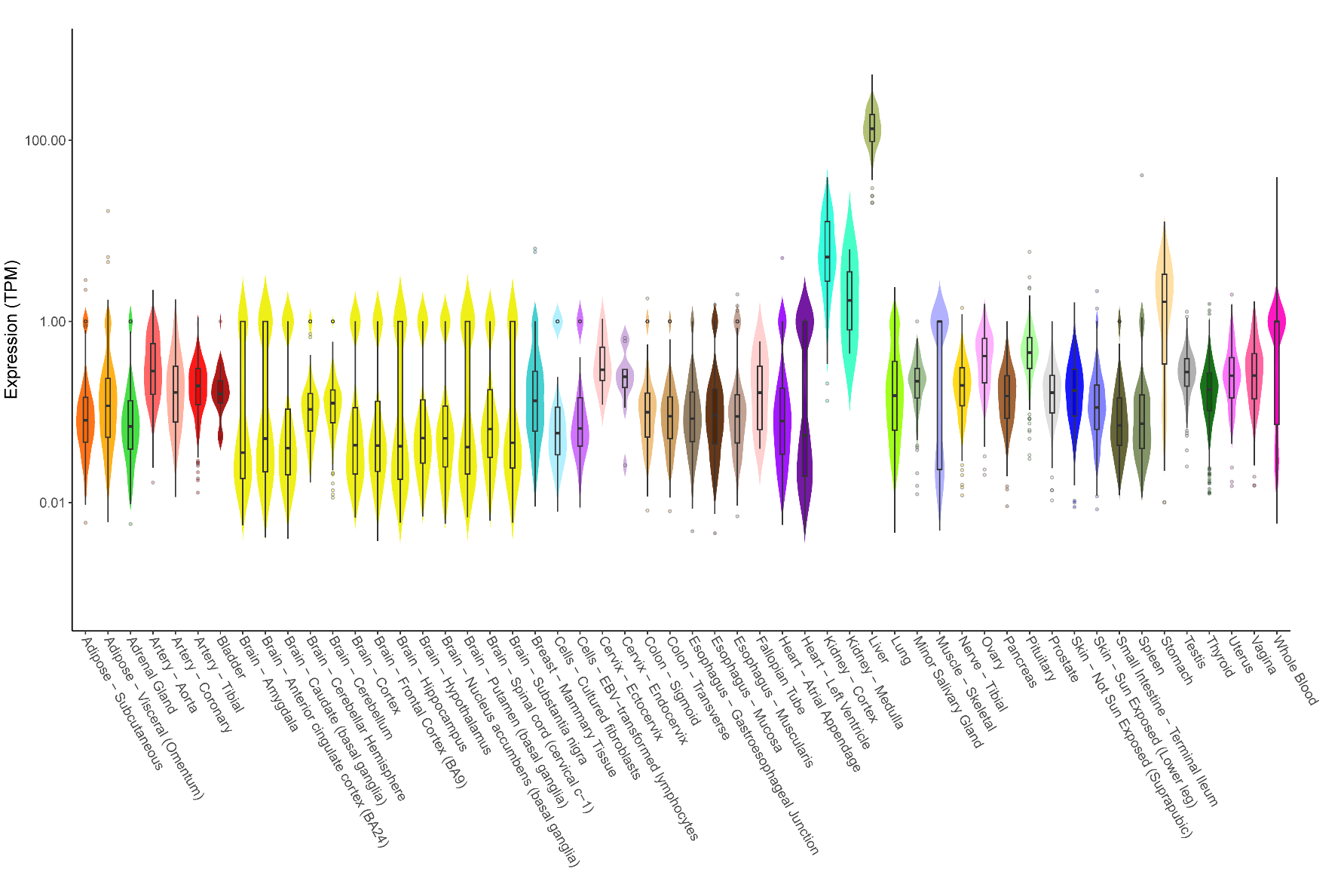
**

**Figure S4. Relative *ANGPTL3* mRNA expression in 49 tissues in GTEx-V8 datasets**

The figure represents the relative mRNA expression levels of the ANGPTL3 gene normalized across 49 different human tissues from the GTEx-V8 datasets.

Abbreviations: TPM, transcripts per million; ANGPTL3, angiopoietin-related protein 3.

**
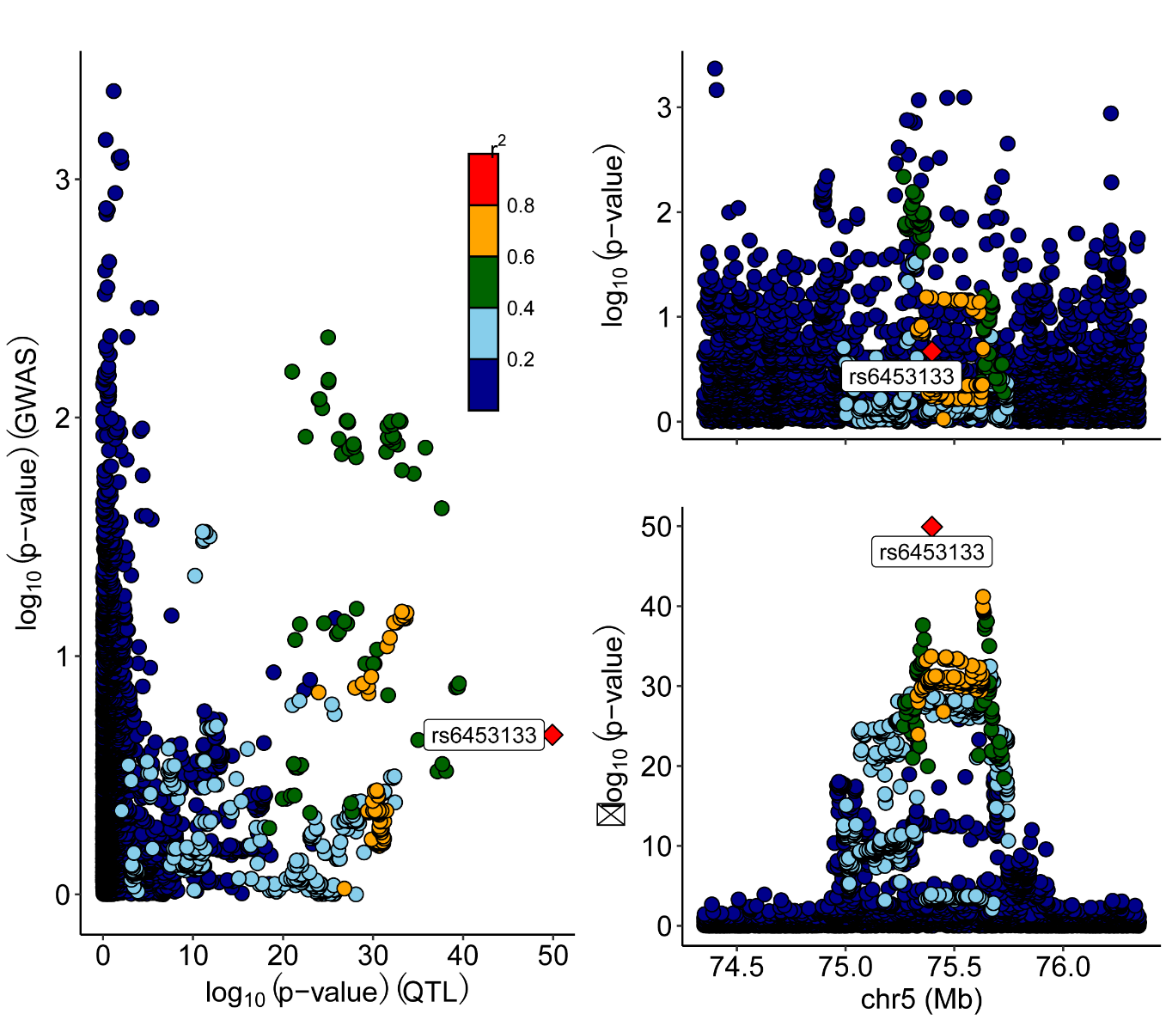
**

**Figure S5. Colocalization analysis of the cis-eQTL for *HMGCR* level in blood tissue and osteonecrosis risk**

rs6453133 is shown as a red diamond. The lead SNP is shown with an arrow. Each dot represents an SNP at the LPL locus. The dots in the scatter plots are colored according to their linkage disequilibrium to the colocalization lead variant. The P values of the SNPs in the HMGCR locus were extracted from the GTEx eQTL data in blood tissue and from the osteonecrosis GWAS. Abbreviations: SNP, single-nucleotide polymorphisms; HMGCR, 3-hydroxy-3-methylglutaryl coenzyme A reductase

**
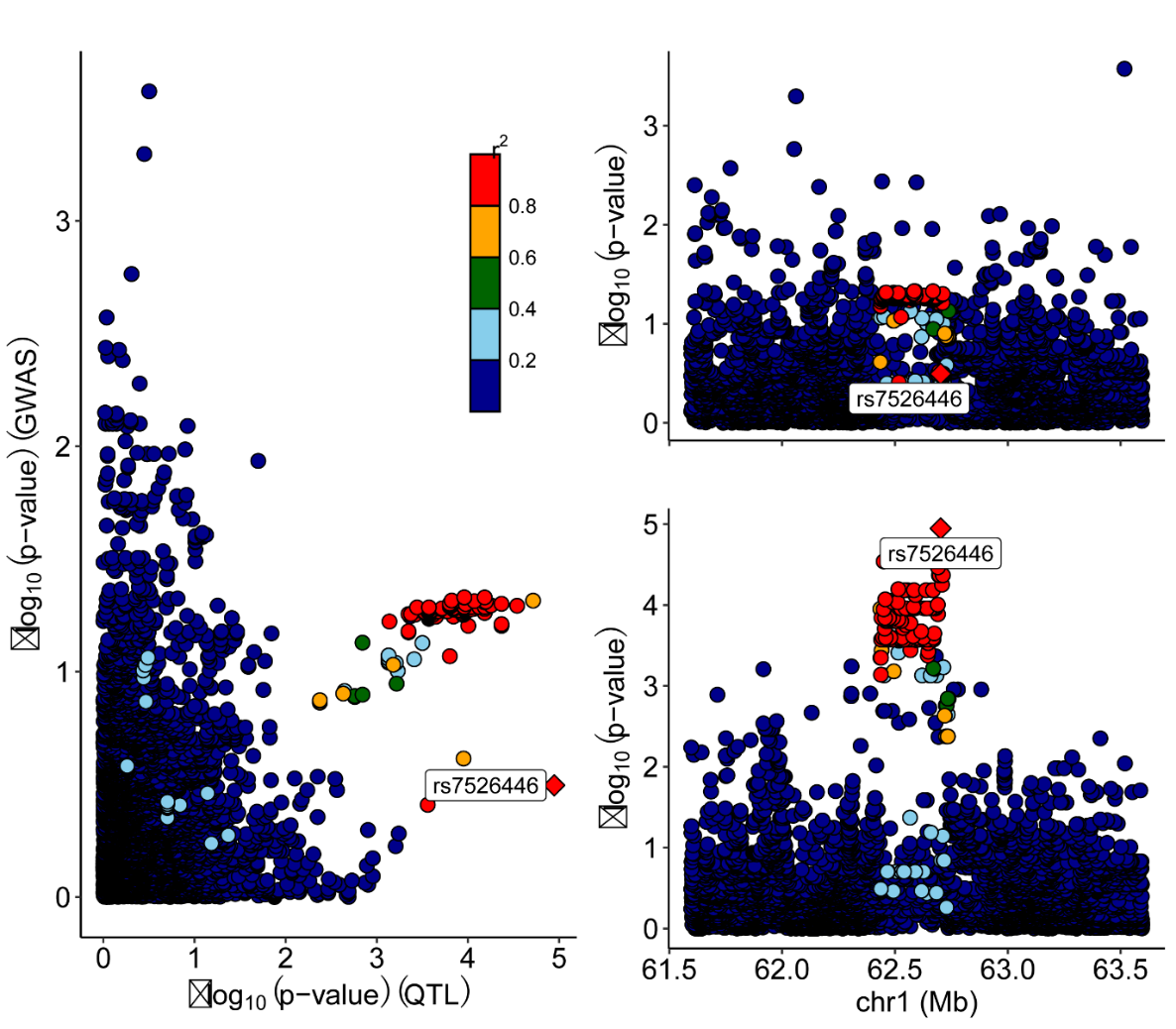
**

**Figure S6. Colocalization analysis of the cis-eQTL for** ***ANGPTL3* level in liver tissue and osteonecrosis risk**

rs7526446 is shown as a red diamond. The lead SNP is shown with an arrow. Each dot represents an SNP at the LPL locus. The dots in the scatter plots are colored according to their linkage disequilibrium to the colocalization lead variant. The P values of the SNPs in the ANGPTL3 locus were extracted from the GTEx eQTL data in liver tissue and from the osteonecrosis GWAS. Abbreviations: SNP, single-nucleotide polymorphisms; ANGPTL3, angiopoietin-related protein 3.
