# Supplementary material for: Statins Regulate Stem Cell Growth Factor‐β to Balance Osteogenesis and Adipogenesis in Mesenchymal Stem Cells, Endowing Anti‐Osteonecrosis Effects
Source: J Cell Mol Med. 2025 Nov 26;29(22):e70967. doi: 10.1111/jcmm.70967 (PMC12648295; doi:10.1111/jcmm.70967)
Supplement: Supplementary file 2 — Data S1: jcmm70967‐sup‐0002‐DataS1.docx. [file JCMM-29-e70967-s002.docx]

### Supplementary materials and methods

### **Micro‐CT analysis**

The femoral heads fixed with 4% paraformaldehyde were analyzed by Bruker Skyscan1276 (Bruker, Belgium). After scanning, analysis data for trabecular bone in the secondary ossification region (the area above the epiphyseal line and below the cartilage) of the femoral head, including trabecular thickness (Tb.Th), trabecular separation (Tb.Sp), bone volume per tissue volume (BV/TV), and trabecular number (Tb.N), were obtained using the CT-Analyzer software (version 1.20.8.0, SkyScan, Bruker). All procedures were performed by a single well-trained operator.

### **Histological analysis**

After micro-CT scanning, the femoral heads were decalcified by 10% ethylenediaminetetraacetic acid. After decalcification for 4 weeks, the specimens were dehydrated and embedded in paraffin. Subsequently, 5 μm paraffin sections in the coronal plane were made by a microtome (Leica, Biocut, German), and slices were used for the hematoxylin & eosin (H&E) staining and the immumohistochemical staining of SCGF-β and β-catenin. In the subchondral bone area, empty lacunae (including osteocytes exhibiting karyopyknosis) in bone were identified and counted by 2 blinded investigators. In short, 2 blind researchers manually counted the total number of cells and the number of cavities in the bone trabeculae of the secondary ossification area, and the percentage of empty lacunae was calculated.

### **ELISA**

The SCGF-β levels of rat serum and MSCs culture supernatant was detected by using Antibody Pair Buffer Kit (CNB0011, Thermo Fisher, Waltham, USA) and anti-SCGF-β antibody(ab9835, Abcam, Cambridge, UK). The ELISA plate is first coated with 100 µL of coating buffer that has been diluted with anti-SCGF-β antibody, followed by overnight incubation at 4 °C. On the following day, the coating solution is discarded, and the plate is blocked by adding 200 µL of blocking buffer to each well and incubated at room temperature for 1 hour. Standards and samples are prepared by diluting them in Assay Buffer (1X), with 100 µL of each added to the respective wells, then incubated at 37 °C for 2 hours. The plate is washed five times with 300 µL of Wash Buffer (1X), prepared by diluting Wash Buffer (25X) in deionized water. Each well receives 100 µL of samples, diluted in Assay Buffer (1X), followed by incubation at 37 °C for 1 hour, and then another five washes. Following this, 100 µL of HRP-conjugated secondary antibody, diluted in Assay Buffer (1X), is added to each well and incubated at 37 °C for 1 hour, followed by five more washes. For color development, 100 µL of the Stabilized Chromogen is added to each well and incubated in the dark for 20 min. The reaction is stopped by adding 100 µL of Stop Solution to each well, and absorbance is read at 450 nm using a microplate reader. All reagents are brought to room temperature before use, and appropriate protective gear is worn, especially when handling the Stop Solution.

### **Oil Red O staining**

Cells were fixed with 4% paraformaldehyde for 30 min, rinsed with water and then stained with Oil Red O solution (six parts of saturated Oil Red O dye in isopropanol plus four parts of water) for 30 min. The cells were washed with water to remove excessive dye. Lipid accumulation in cells was observed under the light microscope. The Oil Red O dye was eluted with 100% isopropyl alcohol, and the absorbance was measured at 500 nm.

### **Alizarin red staining**

Cells are first fixed by adding 4% paraformaldehyde for 15 min at room temperature. After fixation, the paraformaldehyde is removed, and the cells are washed three times with distilled water. The cells are then stained by adding 2% Alizarin Red S solution (pH 4.2) and incubating for 20 min at room temperature. After staining, the Alizarin Red S solution is removed, and the cells are washed three times with distilled water to remove any unbound stain. The stained cells are then visualized under a microscope to assess calcium deposits.

### **Immunofluorescence (IF)**

Cells were fixed with 4% paraformaldehyde for 10-15 min. Next, cells were permeabilized with 0.1% Triton X-100 for 5-10 min. After permeabilization, cells were blocked with 5% normal goat serum at room temperature for 30 min. Subsequently, cells were incubated with the SCGF-β antibody overnight at 4°C. Following primary antibody incubation, cells were incubated with a fluorescently labeled secondary antibody for 1 hour at room temperature in the dark. After secondary antibody incubation, cells were stained with DAPI for 5 min at room temperature. Finally, cells were mounted with an anti-fade mounting medium and observed using a fluorescence microscope to record staining results.

### **Real-time quantitative PCR (RT-qPCR)**

Total RNA was isolated from tissues and cells using RNAiso Plus (TaKaRa, Beijing, China). The concentration of RNA was determined, and 500 ng of RNA was used to reversely transcribed into cDNA using PrimeScript^TM^ RT reagent Kit with gDNA Eraser (TaKaRa, Beijing, China). One microliter cDNA was used to perform real-time PCR in 20 μl reaction mixtures with TB Green Premix Ex Taq^TM^ Ⅱ (TaKaRa, Beijing, China) using a CFX96 real-time PCR system (Bio-Rad, Hercules, CA, USA). The PCR reaction conditions were as follows: initial denaturation at 95 °C for 10 min followed by 30 cycles at 95 °C for 1 min, annealing at 58 °C for 1 min, extension at 72 °C for 1 min and final extension at 72 °C for 5 min. The relative amount of RNA was quantified using the comparative cycle threshold (*C*_T_) (2^−ΔΔ^*^C^*^T^) method. β-Actin was used as internal controls. The results were expressed as folds of control.

### **Western blot (WB)**

Protein extractions of cells were separated using RIPA lysis buffer (P0013B, Beyotime Biotechnology, China). Protein concentration was determined using BCA method (P0012, Beyotime Biotechnology, China). Equal volumes of protein extraction and loading buffer were mixed and separated using SDS-PAGE on 10% polyacrylamide gels. The separated samples were transferred onto polyvinylidene fluoride (PVDF) membranes. After blocking in 5% fat-free milk for 1 h at room temperature, the membranes were incubated with primary antibodies at 4 °C overnight. After washing four times, the membranes were incubated in the appropriate HRP-conjugated secondary antibody at 37 °C for 30 min. Protein bands were detected using chemiluminescent reagents according to the manufacturer's protocol and observed using an image analyzer Quantity One System (Bio-Rad).
